# Lipoprotein‐associated phospholipase A2: The story continues

**DOI:** 10.1002/med.21597

**Published:** 2019-05-29

**Authors:** Fubao Huang, Kai Wang, Jianhua Shen

**Affiliations:** ^1^ State Key Laboratory of Drug Research Shanghai Institute of Materia Medica (SIMM), Chinese Academy of Sciences Shanghai China; ^2^ School of Pharmacy University of Chinese Academy of Sciences Beijing China

**Keywords:** fragment‐based lead discovery, inflammation, inhibitors, lipoprotein‐associated phospholipase A2, vascular diseases

## Abstract

Inflammation is thought to play an important role in the pathogenesis of vascular diseases. Lipoprotein‐associated phospholipase A2 (Lp‐PLA2) mediates vascular inflammation through the regulation of lipid metabolism in blood, thus, it has been extensively investigated to identify its role in vascular inflammation‐related diseases, mainly atherosclerosis. Although darapladib, the most advanced Lp‐PLA2 inhibitor, failed to meet the primary endpoints of two large phase III trials in atherosclerosis patients cotreated with standard medical care, the research on Lp‐PLA2 has not been terminated. Novel pathogenic, epidemiologic, genetic, and crystallographic studies regarding Lp‐PLA2 have been reported recently, while novel inhibitors were identified through a fragment‐based lead discovery strategy. More strikingly, recent clinical and preclinical studies revealed that Lp‐PLA2 inhibition showed promising therapeutic effects in diabetic macular edema and Alzheimer's disease. In this review, we not only summarized the knowledge of Lp‐PLA2 established in the past decades but also emphasized new findings in recent years. We hope this review could be valuable for helping researchers acquire a much deeper insight into the nature of Lp‐PLA2, identify more potent and selective Lp‐PLA2 inhibitors, and discover the potential indications of Lp‐PLA2 inhibitors.

## 1 INTRODUCTION

An increasing number of patients are currently suffering from a variety of vascular diseases, which is a general term for a large class of diseases, including atherosclerosis, peripheral arterial diseases, cerebrovascular diseases (CVDs), aneurysms, and retinopathy.^1^ Due to their wide prevalence and high mortality, vascular diseases have emerged as major public health issues.^2^, ^3^ Although the pathogeneses of vascular diseases are interconnected, inflammation is increasingly understood to play a pivotal role during the disease progression.^4^, ^5^, ^6^ Inflammation is a critical component of the human immune response with double‐edged defense mechanism. Initially, it can protect human physiological homeostasis against infection and tissue damage and be timely terminated when the infectious agents are removed or the initial tissue injuries are repaired. However, failure to resolve the inflammatory response to normal homeostasis may lead to tissue dysfunction and other detrimental consequences.^7^ In regard to vascular inflammation, the unresolved inflammatory response could induce vascular endothelial dysfunction, which is primarily responsible for the occurrence and progression of vascular diseases.^5^, ^8^, ^9^ Therefore, targeting inflammatory pathways, such as upregulating the anti‐inflammatory pathway, downregulating the proinflammatory pathway, and promoting the inflammatory resolution, is considered a promising strategy to treat the vascular diseases.^10^

**Figure 1:**
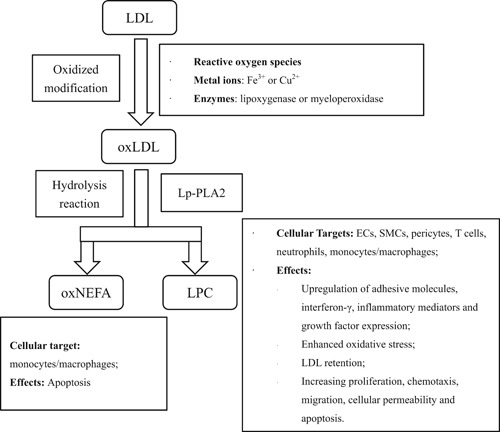
The proinflammatory role of Lp‐PLA2 during LDL oxidization. This figure illustrates the proinflammatory effects of Lp‐PLA2 at cellular level, focusing on the biological activities of its productions. Herein, the adhesive molecules include intercellular adhesive molecule 1 and vascular cell adhesive molecule 1, and the inflammatory mediators comprise the cytokines and proteases.^13^, ^14^ EC, endothelial cell; LDL, low‐density lipoprotein; LPC, lysophosphatidylcholine; Lp‐PLA2, lipoprotein‐associated phospholipase A2; NEFA, nonesterified fatty acid; SMC, smooth muscle cell

Lipoprotein‐associated phospholipase A2 (Lp‐PLA2) belongs to group VII of the PLA2 superfamily, which currently comprises of six types and is divided into 16 unique groups.^11^ This enzyme is principally secreted by macrophages and circulates in the blood in the form of a complex with low‐density lipoprotein (LDL) and high‐density lipoprotein (HDL).^12^ Lp‐PLA2 was originally named plasma platelet‐activating factor acetylhydrolase (pPAF‐AH), due to its hydrolytic action on platelet‐activating factor (PAF).^11^ In addition to PAF, Lp‐PLA2 could hydrolyze oxidized LDL into two bioactive products, lysophosphatidylcholine (lysoPC) and oxidized nonesterified fatty acids (oxNEFAs).^13^ LysoPC seemingly represents the majority of Lp‐PLA2‐derived proinflammatory effects. It can target endothelial cells (ECs), smooth muscle cells (SMCs), monocytes/macrophages, T cells, and neutrophils; affect cell viability, homing of inflammatory cells, and functional responses of ECs and SMCs; and induce oxidative stress and immune responses (Figure 1).^13^ Furthermore, lysoPC was recently demonstrated to induce the pericytes loss in the central nervous system (CNS), which is indicative of an injured blood‐brain barrier (BBB).^14^ Because of the proinflammatory effects and high distribution in blood, together with the observation that elevated plasma Lp‐PLA2 levels were associated with a number of vascular diseases in epidemiological studies,^15^, ^16^, ^17^, ^18^ the effect of Lp‐PLA2 inhibition on vascular diseases have been assessed in animal models and clinical trials. Unfortunately, darapladib, which was discovered by GlaxoSmithKline (GSK) and is the most advanced Lp‐PLA2 inhibitor in clinical trials, missed the primary endpoints in two phase III trials in atherosclerosis patients in 2014.^19^, ^20^ Although these failures dim the future of anti‐inflammatory drug discovery for atherosclerosis, favorable results of applying Lp‐PLA2 inhibitors to diabetic macular edema (DME) and Alzheimer's disease (AD) have been consistently reported over the last few years.^21^, ^22^

In the past decade, a number of reviews have been published with respect to the structure,^23^ genetic aspects,^24^ biological roles,^25^ disease implications,^12^, ^26^, ^27^ and specific inhibitors of Lp‐PLA2.^28^ In addition, Lp‐PLA2 has been reviewed as a part of the PLA2 superfamily^11^, ^29^ or PAF‐AHs.^30^, ^31^ Nevertheless, in this review, we comprehensively summarize the Lp‐PLA2 studies on the gene, regulation of expression, biological functions, indications, crystal structures, and inhibitors. In addition, we highlighted recent advances in Lp‐PLA2 and Lp‐PLA2 inhibitors. The newly established crystal structures of Lp‐PLA2 after soaking with different inhibitors and the identification of new binding pockets may advance the discovery of potent and selective inhibitors.^32^, ^33^, ^34^, ^35^ Notably, the successful application of fragment‐based lead discovery (FBLD) in developing Lp‐PLA2 inhibitors provides a referential practice for other targets’ lead discovery. Furthermore, the new knowledge of the role of Lp‐PLA2 in certain diseases^36^, ^37^ and the substantial amounts of data generated from clinical trials^38^ might help extend the applied range of Lp‐PLA2 as a therapeutic target. Therefore, we hope that this updated, comprehensive review can help the interested researchers become better acquainted with Lp‐PLA2 and attract more attention to Lp‐PLA2 research.

## 2 BIOCHEMICAL PROPERTIES AND STRUCTURAL CHARACTERISTICS

### 2.1 Biochemical properties

The human plasma Lp‐PLA2 is a 45 kDa hydrophobic protein that functions in the absence of calcium. It possesses the ability to catalyze hydrolysis at the *sn*‐2 position of phospholipids, while sequence analysis revealed a *GXSXG* motif, the characteristic fingerprint for neutral lipases and serine esterases, in the primary structure of Lp‐PLA2.^25^ Consequently, Lp‐PLA2 exhibits characteristics of both PLA2s and neutral lipases. Even though hematopoietic cells might be chiefly responsible for the circulating Lp‐PLA2 levels, a number of tissues, such as liver cells,^39^ aorta cells,^40^ and adipocytes,^41^ seem to be additional sources. After secretion, Lp‐PLA2 circulates by binding to lipoproteins in plasma, whereby LDL and HDL carry 70% to 80% and 20% to 30% of the total plasma activity, respectively.^11^ Lp‐PLA2 acts on the substrate in the aqueous phase, as no signs of interfacial activation are associated with Lp‐PLA2.^42^ However, given that the surfaces of both LDL and HDL are enriched with phospholipids, we cannot rule out the possibility of Lp‐PLA2 binding to its substrates from the lipid membrane phase, which is the case for all typical membrane‐associating PLA2 enzymes.^11^ Indeed, this speculation was subsequently supported by two studies, which implied that the substrates could enter the catalytic center of Lp‐PLA2 from lipoprotein particles.^43^, ^44^ Nevertheless, this hypothesis remains to be established, as supporting evidence is limited.

The susceptibility to oxidative inactivation is another odd property of Lp‐PLA2, in view of its elevated expression in response to oxidative stress. The inhibition of Lp‐PLA2 activity, which apparently results from both physiological (heavy metals and oxygen radicals) and nonphysiological (cigarette smoke) oxidants, may involve irreversible modification of key amino acid residues. Peroxynitrite, one of the key oxidants produced in cellular oxidation in vivo, was identified to inactivate Lp‐PLA2.^45^ Using site‐directed mutagenesis, MacRitchie et al^46^ revealed that a primary target of oxidation in the protein was Met117, which was exposed on the protein surface; its oxidation would not only result in enzymatic inactivation but also affect the association with LDL. In addition, the tyrosine nitration of Tyr307 and Tyr335 also moderately contributed to the inactivation of the enzyme. Very recently, Gurung et al^45^ used molecular dynamics simulation and essential dynamics in tandem with a molecular docking approach to elucidate the effect of the structural alteration of Lp‐PLA2 upon the oxidation of the aforementioned three amino acids. The results revealed that Met117 oxidation induced enhanced flexibility, decreased compactness in the oxidized state, and insubstantial binding to the substrate PAF.^45^ In addition to the decreased binding affinity of the substrate and high flexibility, nitration of Tyr307 and Tyr335 also led to the disorientation of the catalytic triad and a reduction in the molecular interactions of NT‐Tyr307 and NT‐Tyr335 with other residues of the protein.^47^ Even though these findings offered some plausible mechanism for the reduction in enzymatic activity of Lp‐PLA2 under oxidative stress, further insights into the molecular mechanisms are anticipated through studies using other biophysical techniques such as nuclear magnetic resonance, cryoelectron microscopy and X‐ray crystallography.

### 2.2 Substrate specificity

At the time of discovery, Lp‐PLA2 was found to be capable of hydrolyzing PAF, a phosphatidylcholine containing an acetate group at the *sn*‐2 position. Subsequent studies demonstrated that Lp‐PLA2 possessed broad substrate specificity. A distinguishing feature of Lp‐PLA2, compared with other members of the phospholipase A2 superfamily, is its specific recognition of the type of *sn*‐2 residue to be hydrolyzed. Compared with the short acetyl chains at the *sn*‐2 position of PAF, the extended *sn*‐2 residue of PAF analogs are hydrolyzed by Lp‐PLA2 with decreased efficiency; for instance, the C5 homolog is 60% as efficient as PAF, the C6 homolog is 20% as efficient, and the C9 homolog is only 2% as efficient. However, if the *sn*‐2 residue of phospholipids contains a ω‐terminal oxo function, such as an aldehyde or a carboxyl, this length restriction is greatly relaxed, and *sn*‐2 acyl chains of up to nine carbon atoms in length can be tolerated.^11^ In addition, Lp‐PLA2 could efficiently hydrolyze short‐chain diacylglycerols, triacylglycerols, and acetylated alkanols, signifying that a polar headgroup might not be required for Lp‐PLA2 substrates. Furthermore, the enzyme shows a weak specificity at the *sn*‐1 position, as it could not distinguish ester from ether at the *sn*‐1 position of PAF or PAF analogs.^48^ Apparently, glycerides with a *sn*‐2 ester and a reasonably hydrophobic *sn*‐1 residue would be efficient substrates of Lp‐PLA2. Interestingly, Lp‐PLA2 display phospholipase A1 activity that specifically removes the *sn*‐1 acyl group of phospholipids under certain conditions.^31^

Truncated phospholipids with shortened and oxidized *sn‐2* residues are mainly generated via the oxidative attack on phospholipid components of cellular membranes and lipoproteins. Considering the substrate selectivity, the identification of truncated oxidized phospholipids (oxPLs) as substrates of Lp‐PLA2 is comprehensible.^49^ Two analyses indicated that in vitro incubation of LDL with copper sulfate will generate oxidized modified LDL, in which lysoPC appeared to be the major product. However, its generation was strongly suppressed by the treatment of human LDL with a Lp‐PLA2 inhibitor, suggesting that Lp‐PLA2 activity is critical for lysoPC generation in LDL.^50^, ^51^ Further, mass spectrometry (MS)‐based analyses were carried out to investigate the changes in the compositions of native and copper‐oxidized LDL phosphatidylcholines in the presence and absence of an Lp‐PLA2 inhibitor. As a result, the major oxidized PC (oxPC) species that accumulated after inhibitor treatment were a cleaved oxPC, 1‐palmitoyl‐2‐(9‐oxo‐nonanoyl) PC, and a long‐chain oxPC species with two double bonds, indicating possible hydrolysis by Lp‐PLA2.^51^ Both of these studies established that truncated oxPC was more efficient than long‐chain oxPC as a substrate for Lp‐PLA2. With a better understanding of the physiological functions of Lp‐PLA2, more oxidized phospholipids with complicated substituents at the *sn*‐2 position were defined as substrates for Lp‐PLA2. For example, peroxidized 1‐palmitoyl‐2‐oleoyl phosphatidylcholine,^52^ a phospholipid hydroperoxide, and esterified F_2_‐isoprostanes^53^ were also identified to be hydrolyzed by Lp‐PLA2, though both harbor a long carbon chain at the *sn*‐2 position. Recently, Buland et al^54^ discovered that Lp‐PLA2 could hydrolyze the oxidized cardiolipin (CLox) in the extracellular space, which resulted from the oxidation of the polyunsaturated mitochondrial phospholipid cardiolipin (CL). Notably, the accumulation of lysoCL and oxidized octadecadienoic acid metabolites in extracellular spaces may lead to an impairment of pulmonary endothelial barrier function and necrosis during the hydrolysis of CLox by Lp‐PLA2.^54^

In addition, Lp‐PLA2 can utilize oxidized phosphatidylserine species (PSox) as substrates. In this context, Tyurin et al^55^ found that Lp‐PLA2 was highly effective in catalyzing the hydrolysis of diverse nontruncated and truncated peroxidized species of PSox formed as a result of cytochrome *c*‐ and H_2_O_2_‐driven enzymatic oxidation of phosphatidylserines. As in the case of the oxidized PC species, Lp‐PLA2 was comparatively effective in the catabolism of oxidatively fragmented PSox species occurring at the *sn*‐2 position. Further computer modeling revealed that the preferable substrates might be PSox species whose *sn*‐2 ester bonds were in proximity with Ser273 and oxygenated functional groups were hydroxy or hydroperoxy, particularly, at the C9 position of the *sn*‐2 chain.^55^ Furthermore, Lp‐PLA2 possesses transacetylase activity, that is, it can transfer acetyl from PAF to ether‐ or ester‐linked lysophospholipids.^31^

In general, oxPC, PAF and its analogs are the major substrates for Lp‐PLA2, while PSox and other types of oxPL constitute the minor ones. In both PC and PS, the oxygenated long‐chain fatty acid residues may be the dominant products of oxidation and are less efficient Lp‐PLA2 substrates, while truncated oxidized species with low abundance are preferentially hydrolyzed by Lp‐PLA2. Interestingly, Greenberg et al^56^ observed that the *sn*‐2 fatty acid moiety of oxidatively fragmented phospholipids no longer remained in the hydrophobic core of the membrane, but rather protruded into the aqueous phase. Along with the finding that the enzyme possibly accessed its substrates from the aqueous phase, Kono et al^31^ speculated that the substrate specificity of Lp‐PLA2 might be related to the conformational orientation of the oxidized *sn*‐2 fatty acid species in the membrane bilayer. In addition, oxPL species were shown to be chemically linked to naturally occurring human proteins such as apolipoprotein (a) and plasminogen; hence, the covalent lipid adducts might affect the hydrolysis of Lp‐PLA2 by accessibility to the *sn*‐2 acyl group. Since two studies obtained inconsistent results regarding whether these covalent adducts could protect oxPL from being metabolized by Lp‐PLA2,^57^, ^58^ further studies are essential to address this issue.

### 2.3 The interaction of Lp‐PLA2 with lipoprotein particles

As mentioned previously, Lp‐PLA2 circulates in the plasma by binding to LDL and HDL, preferentially the apoB‐containing LDL and apoA‐I‐containing HDL. Specifically, Lp‐PLA2 prefers to associate with the small dense or electronegative LDL fractions and HDL3c, also denoted as very high‐density lipoprotein‐1 subfraction (VHDL‐1). Low concentrations of Lp‐PLA2 can bind to lipoprotein (a) (Lp (a)) and very low‐density lipoprotein (VLDL), while the enzyme has higher affinity to Lp (a) than to LDL in individuals with elevated Lp (a) levels. Owing to its low abundance, only a small proportion of circulating lipoproteins carry Lp‐PLA2, even in the most enriched subfractions.^23^ Interestingly, the enzyme can transfer between LDL and HDL particles,^59^ and the variation in the distribution of Lp‐PLA2 among lipoproteins was observed in patients with paroxysmal atrial fibrillation, chronic kidney disease (CKD), hyperlipidemia, and coronary artery disease (CAD).^60^ The proposal that HDL‐bound Lp‐PLA2 is antiatherogenic while LDL‐bound enzyme has the opposite effect, together with the diverse metabolic behavior between the LDL particles that carry Lp‐PLA2 and those that lack this enzyme, apparently indicates that the dynamic distribution of Lp‐PLA2 is likely to influence the pathophysiology, clinical significance, and behavior of pharmacological intervention in humans.^12^

To identify the specific regions of Lp‐PLA2 involved in binding to LDL and HDL, a number of studies were performed based on the apo form of Lp‐PLA2 structures. Consequently, two short α‐helices rich in hydrophobic residues are identified to form the interface binding surface (i‐face) and presumably insert into the interfacial region of the membrane. Specifically, the residues Tyr205, Trp115, Leu116, and to a lesser extent, Met117, which are part of the N‐terminal α‐helix, are probably essential for binding to LDL,^23^ whereas a part of the C‐terminal α‐helix (His367 to Lys370) accounts for the association with HDL.^61^ Additionally, other factors can also affect the association between lipoproteins and Lp‐PLA2. For example, N‐linked glycosylation of plasma Lp‐PLA2 was shown to hinder its binding to HDL in humans and Asn423 and 433 might be involved in this process.^62^ Moreover, charged amino acids may also be potentially involved in the interaction with lipoproteins. The preference of Lp‐PLA2 for small dense LDL, electronegative LDL and Lp(a) was proposed to be mediated by the altered conformation of apoB100, the major apolipoprotein present on LDL, in which the exposure of additional regions with positively charged residues may electrostatically interact with the negatively charged acidic patch of Lp‐PLA2. In addition, specific basic patch amino acids could interact with either the apoproteins or the phospholipid component of LDL and HDL particles. As an example, the charged state of His114 present on the binding surface likely determines the pH‐dependent binding of Lp‐PLA2 to LDL. Seemingly, the interactions between residues of Lp‐PLA2 and LDL or HDL are electrostatic in nature, and these electrostatic interactions may play a key role in modulating the distribution of the enzyme between lipoproteins.^23^

The alteration of the structural properties of lipoproteins also affects the interaction with Lp‐PLA2. Pande et al^43^ demonstrated that increasing the amount of cholesterol in the phospholipid membranes has a significantly dissimilar effect on membrane binding, the membrane penetration, and the activity of purified Lp‐PLA2, which varied with the diverse composition of membrane vesicles.^43^ In addition, different species of oxPLs induce distinctive changes in the physicochemical properties of the lipid particles/membranes containing them, presumably depending on the chemical nature of polar functional groups in their oxidized fatty acyl chain. Therefore, the presence of oxPLs in LDL is predicted to regulate the recruitment and the catalysis of Lp‐PLA2.

Notably, the nature of Lp‐PLA2 distribution among lipoproteins is species‐dependent. In species that express lower concentrations of LDL, such as guinea pigs, rats, and mice, Lp‐PLA2 is carried exclusively by HDL in plasma. A structure‐based study revealed that the lack of Trp and Leu in mouse Lp‐PLA2 at the positions corresponding to Trp115 and Leu116 in the human protein might be responsible for the varying distribution. Moreover, the levels of LP‐PLA2 vary extensively among mammalian species, and basal Lp‐PLA2 activity in humans is 10‐fold lower than that in mice. These discrepancies might lead to difficulty in reproducing the results from animal experiments in humans, especially allowing for the different pathophysiological functions of Lp‐PLA2 within diverse lipoproteins.^60^ Thus, continued efforts to elucidate the physiological consequences resulting from these differences among species are essential. Another issue that needs to be taken into account is that Lp‐PLA2 possibly utilizes the same regions to interact with both LDL and HDL, as determined based on its structure and hydrogen‐deuterium exchange experiments.^44^ Consequently, further studies to identify the HDL and LDL regions should be conducted with both lipoprotein particles in parallel, considering the fact that previous results were obtained from binding experiments focused exclusively on only one lipoprotein particle.

### 2.4 Structural characteristics of Lp‐PLA2

**Figure 2:**
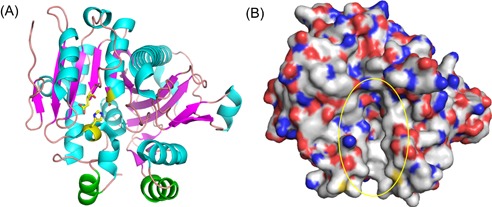
The crystal structure of apo Lp‐PLA2. The figure was prepared using the program PyMOL. A, The cartoon model of apo Lp‐PLA2 crystal structure. The α helix, β sheet, and loop are shown with cyan, magenta, and salmon, respectively. The green regions represent two lipoprotein‐binding helices (residues 114‐126 and 362‐369). The catalytic triad (Ser273, His351, and Asp296) is shown with light green sticks. B, The surface of apo Lp‐PLA2 crystal structure. The carbon, oxygen, nitrogen, and sulfur atoms are shown with gray, red, blue, and yellow, respectively. The yellow circle indicates the ligand binding sites (PDB: 3D59). Lp‐PLA2, lipoprotein‐associated phospholipase A2 [Color figure can be viewed at wileyonlinelibrary.com]

Lp‐PLA2 is a secreted enzyme whose first 17 residues (Met1 to Ala17) presumably constitute a hydrophobic signal peptide. The N‐terminus of endogenous Lp‐PLA2 protein is heterogeneous and possesses Ser35, Ile42, or Lys55, while the C‐terminus contains Asn441 and includes two potential N‐linked glycosylation sites at the residues Asn423 and Asn433.^63^ The primary structure of Lp‐PLA2 is unique and homologous to a conserved Gly‐X‐Ser‐X‐Gly motif found in serine esterases, including many lipases.^11^ Furthermore, elegant crystallographic analyses performed on *Escherichia coli*‐expressed enzyme with 383 nonglycosylated residues (47‐429) revealed that the enzyme contains the classic lipase/esterase α/β‐hydrolase fold (Figure 2). The ligand‐free crystal belongs to the space group *C2* and contains two structurally similar subunits A and B in the asymmetric unit^64^; the structure between the A and B subunits is not significantly different, with an overall 0.3 Å C_α_ RMSD (root mean square error). Since the enzyme is assumed to function as a monomer, this dimerization is merely due to crystal packing and plays no functional or physiological role. Noticeably, an atypical cis‐peptide bond exists between Phe72 and Asp73 in each subunit of both the structures reported and the presence of this bond is further supported by the distinct electron density of this region.^65^

**Figure 3:**
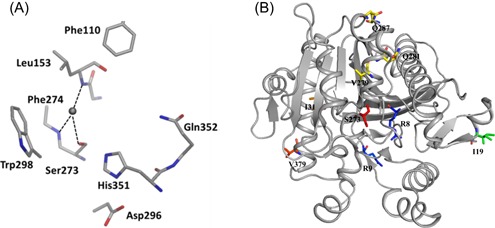
A, The catalytic triad and oxyanion hole of Lp‐PLA2. B, The locations of selected variants based on the apo Lp‐PLA2 structure. A, Cited from the study of Woolford et al.^35^ B, The Ser273 is shown with red sticks and the mutant sites are labeled. The figure was prepared using the program PyMOL (PDB: 3D59). Lp‐PLA2, lipoprotein‐associated phospholipase A2 [Color figure can be viewed at wileyonlinelibrary.com]

A catalytic triad comprising Ser273, His351, and Asp296 and an oxyanion hole constituted by the backbone NH groups of Leu153 and Phe274 make up the binding site of Lp‐PLA2 (Figure 3A). In the active site, nucleophilic Ser273 is located at the N‐terminus of a core α‐helix and Asp296 and His351 are positioned suitably to activate Ser273 for catalysis (Figure 2A). The Leu153 and Phe274 of the oxyanion hole stabilize the incipient negative charge present on the tetrahedral intermediate produced during the ester hydrolysis of a substrate. As observed in other lipases, the catalytic triad is located within a hydrophobic pocket that is oriented toward its substrate, facing the aqueous phase and lying just above the interface (Figure 2B). As a result, the active site would theoretically allow substrates to enter from the aqueous phase as well as from both lipoprotein particle carriers. Additionally, the binding pocket of Lp‐PLA2 is large enough to accommodate bulky substrates, while its predilection for substrates with short and polar *sn*‐2 chains is possibly due to their aqueous phase solubility, which may influence their accessibility to the active site.^42^ Furthermore, the positions of the main single‐nucleotide polymorphism (SNP) sites are shown in Figure 3B. Mutations on residues Q281 and V279 might disrupt the active site, while those on residues R92, V379, and I198 are believed to affect the interactions between Lp‐PLA2 and lipoproteins.

Several disordered residues and side chains exist in the ligand‐free crystal structure and have indistinct or even absent electron densities. For example, seven residues (Ala47 to Gln53) from the N‐terminus and residues 426 to 429 of subunit A and residues 428 to 429 of subunit B from the C‐terminus could not be modeled. Nevertheless, the ordered region of the ligand‐free structure corresponds with the tolerated functional limits of N‐ and C‐terminal truncation formerly determined. Furthermore, His114, Trp115, and Leu116 show weak electron densities in the ligand‐free crystal structure and are predicted to be flexible as a result of the superficial location and predict membrane interactions. In addition, several other surface‐accessible and polar side chains from the ligand‐free crystal structure are disordered and consequently not modeled.^65^

### 2.5 Structural characteristics of Lp‐PLA2 complexed with covalent binding ligands

**Figure 4:**
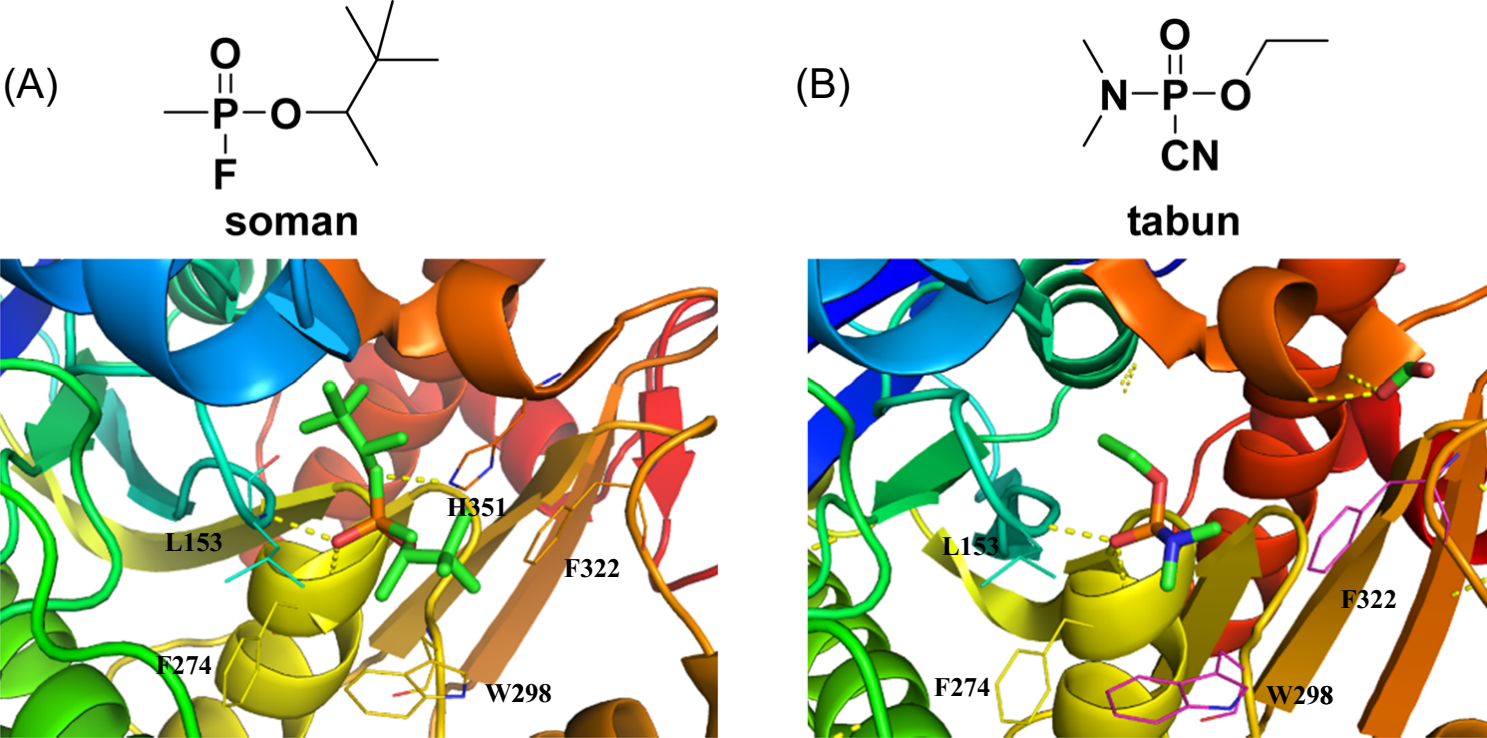
The crystal structure of Lp‐PLA2 bound to (A) soman (PDB: 3F97) and (B) tabun (PDB: 3F98). The figure was prepared using the program PyMOL. Both the P_S_ and P_R_ stereoisomers of soman (A) could covalently bind to Lp‐PLA2, while P_R_ stereoisomer of tabun (B) is preferable to covalently attach to Lp‐PLA2. Lp‐PLA2, lipoprotein‐associated phospholipase A2 [Color figure can be viewed at wileyonlinelibrary.com]

The crystal structures of Lp‐PLA2 were obtained as covalent complexes with organophosphorus (OP) compounds. Five known OP compounds, namely, paraoxon,^65^ *O*,*O*‐diisopropylfluorophosphate (DFP), soman, sarin, and tabun,^64^ were validated to covalently bind at the active site Ser273 by mass spectral data. The crystal structures of these covalent complexes revealed well‐defined differences in electron densities with ordered side‐chain positions for all the residues modeled. The side‐chain positions of these OP complex crystal structures were fundamentally identical to those in the ligand‐free protein structure, as the overall C_α_ RMSD between each OP complex and the ligand‐free structure ranges from 0.25 to 0.5 Å. Apart from the tabun adduct structure containing three subunits, the other four complexed structures were determined to be in the space group *C2* with two subunits in the asymmetric unit.^66^ Furthermore, the other four complexed structures showed similar positions of the active‐site residues and neighboring residues, which was consistent with the tetrahedral intermediate of the reaction mechanism. Nevertheless, the structure of tabun complexed with Lp‐PLA2 had an overall C_α_ RMSD of 0.5 Å compared with the ligand‐free structure, which resulted in differences in the contact between Ser273 and its adjacent residues. In addition to the covalent bond with Ser273, the double‐bonded phospho‐oxygen within the five OPs interacted with the backbone amide of Phe274 and Leu153 through hydrogen bonds located in the oxyanion hole of the enzyme. In the case of the active‐site His351, paraoxon and DFP formed a hydrogen bond with the side chain nitrogen of the residue through the oxygen atoms in the alkoxy portion, which prevented the amino acid from reverting to its unprotonated form. However, sarin and soman had chiral centers at the P atom and showed hydrogen bonding with His351 only in specific P stereoisomers, while tabun, containing a chiral P atom, failed to generate an interaction with His351, probably due to the electronic effect of the *N*,*N*‐dimethyl group displacing the active site His351 away from its native and functional conformation (Figure 4A and 4B). In the complex of Lp‐PLA2 with tabun, good binding stability might be attributed to a CH–π interaction of the *N*,*N*‐dimethyl group with Phe322 and Trp298 of subunit A and Phe51 of subunit C. Notably, the tabun adduct on Lp‐PLA2 additionally showed a stereoselective preference for the P_*R*_ stereoisomer, whereas both the P_*R*_ and P_*S*_ stereoisomers of sarin and soman apparently bind to the enzyme with equal occupancy.^64^

OP compounds prevent the degradation of the neurotransmitter acetylcholine through the inhibition of acetylcholinesterase (AChE), posing a fatal threat to human life. Subsequent to binding AChE, OP compounds undergo a rapid dealkylation process referred to as aging, which irreversibly prevents the enzyme from being reactivated by nucleophiles to regenerate the enzymatic activity. However, if the compounds do not age upon binding, the complex may become reactivated by the attack of an activated water molecule or an activated nucleophile. In addition, numerous serine hydrolases, including Lp‐PLA2, have been shown to be inactivated by OP compounds. Efforts have focused on engineering diverse serine hydrolase enzymes to neutralize or scavenge nerve agents in the plasma before they reach the AChE targets in the peripheral and CNSs. Thus, Lp‐PLA2 seems to be an excellent bioscavenger target due to its presence in human blood.^64^, ^65^

All five crystal structures of Lp‐PLA2 with OP adducts mentioned above were validated to be in the preferred nonaged form, and the interactions revealed by these complexes offer a starting point to develop catalytic bioscavengers. On the basis of the crystal structures of Lp‐PLA2 with the OP adducts mentioned above, Kirby et al^67^ induced a number of histidine mutations near the active site of the enzyme in an endeavor to generate novel OP‐hydrolase activity. As a result, the W298H mutant displayed novel somanase activity with a *k*_cat_ of 5 minutes^−1^ and a *K*_M_ of 590 μM at pH 7.5, representing a catalytic efficiency of 8.4 × 10^3^ M^−1^·min^−1^. Even though the catalytic efficiency remains to be determined in vivo, the accomplishment will encourage more researchers to explore catalytic bioscavengers, which will effectively protect humans from these nerve agents in vivo.^67^

### 2.6 Structural characteristics of Lp‐PLA2 complexed with reversible inhibitors

**Figure 5:**
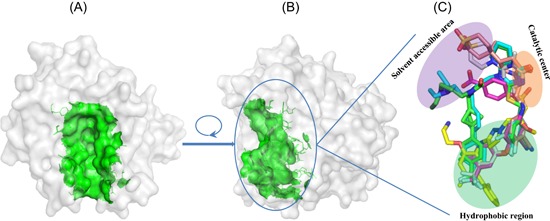
A,B, The known binding sites of reversible inhibitors showing on apo Lp‐PLA2. C, Overview of binding profiles of these inhibitors. The figure was prepared using the program PyMOL. A, Superimposed binding pockets of inhibitors are shown with green surface. Then, they are performed on the structures of apo Lp‐PLA2. B, The view shows the result after a 90° clockwise rotation on the *Y*‐axis from the view shown in (A). C, Generally, reversible inhibitors with known crystal structures share three binding sites within catalytic center, hydrophobic region, and solvent‐accessible area, except compound **5** (yellow) which do not interact with catalytic center. Lp‐PLA2, lipoprotein‐associated phospholipase A2 [Color figure can be viewed at wileyonlinelibrary.com]

In recent years, a number of crystal structures of Lp‐PLA2 in complex with reversible inhibitors have been reported. Similar to OP complex crystal structures, two virtually identical copies of the complex were observed in the asymmetric unit of both structures. Additionally, the frameworks of these reversible inhibitor‐bound complexes were similar to those of the earlier covalent‐bound structures as well as the ligand‐free form. The binding pocket of these inhibitors primarily relies on a gorge that initiates from the catalytic triad and extends to two solvent‐accessible α‐helices (residues 114‐126 and 362‐369), which were validated to participate in binding to the lipoprotein particles LDL or/and HDL (Figure 5A and 5B). These inhibitors perfectly nestled into the binding gorge and usually contained three binding sites, including the catalytic center, hydrophobic region, and solvent‐accessible area (Figure 5C).

**Figure 6:**
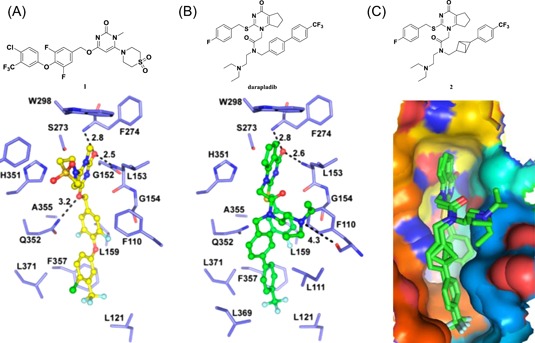
Interactions between Lp‐PLA2 and compound **1** (PDB: 5I8P) (A) and darapladib (PDB: 5I9I) (B). C, Superimposed binding pockets of darapladib and compound **2** (PDB: 5LP1). A,B, Cited from our recent study.^32^ Residues are shown in sticks, and inhibitors are rendered in ball and stick. Distances of two polar atoms that form a hydrogen bond are labeled in Å. Superimposed binding pockets of inhibitors are shown with green surface. Then, they are performed on the structures of apo Lp‐PLA2. The view shows the result after a 90° clockwise rotation on the *Y*‐axis from the view shown in (A). C, The view of superimposed binding surfaces of darapladib and compound **2**. The figure was prepared using the program PyMOL. Lp‐PLA2, lipoprotein‐associated phospholipase A2 [Color figure can be viewed at wileyonlinelibrary.com]

Our group first determined the crystal structures of human Lp‐PLA2 bound with two potent inhibitors, darapladib (Figure 6B) and compound **1** (Figure 6A), which were diffracted to 2.7 and 2.37 Å, respectively. Compound **1** was recently identified as an Lp‐PLA2 inhibitor by our group. The pyrimidinone carbonyl of darapladib and compound **1** interacts with the oxyanion hole in the enzyme through two bonds to the backbone amides of Phe274 and Leu153. In addition, several residues (Leu371, Phe357, Trp298, Leu153, Leu111, and Phe110) display hydrophobic interactions with darapladib. The two benzene rings of the biphenyl moiety form an intramolecular edge‐to‐face π‐stacking interaction with the fluorophenyl ring and extend into a predominantly lipophilic channel toward Phe125. Apart from two conserved H‐bonds, the complex of Lp‐PLA2 and **1** formed a third and unique H‐bond between the oxygen linked to the pyrimidone ring and the side chain of Gln352. The residues that contribute to the hydrophobic interactions are identical to those presented in the Lp‐PLA2 and darapladib complex, except that Gly152 and Phe322 are added while Leu111 and Leu369 are missing. Additionally, an edge‐to‐face π–π interaction is formed between the terminal benzene ring of compound **1** and Phe357. The superimposition of the two complex structures reveals that the bound darapladib and **1** greatly overlap except the diethylamine group of darapladib, as no corresponding group is found in **1**. Further isothermal titration calorimetry measurements demonstrate that the enthalpic effects drive the binding of two inhibitors to Lp‐PLA2, which is consistent with the multiple protein‐ligand interactions revealed by the two complex structures.^32^ Compound **2**, a bioisostere of darapladib, showed improved physicochemical properties compared with darapladib (Figure 6C). The crystal structure of bioisosteric analog **2** in the Lp‐PLA2 protein was solved at 1.9 Å resolution and revealed a binding mode similar to that of darapladib. The replacement of the phenyl ring with a bicyclo[1.1.1]pentane moiety slightly precludes the adjacent trifluorophenyl moiety extending as far toward Leu121 and Phe125, while showing no effect on the main interactions within the oxyanion hole.^68^

**Figure 7:**
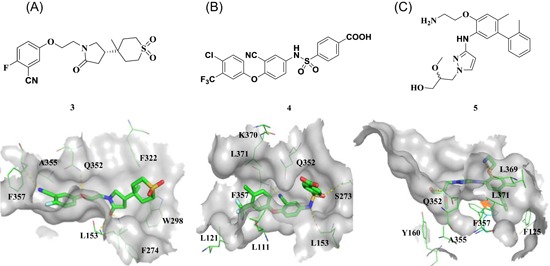
Interactions between Lp‐PLA2 and compound **3** (A) (PDB: 5LZ9), **4** (B) (PDB: 5YEA) and **5** (C) (PDB: 5JAU). Interacting residues are shown in green lines, and inhibitors are rendered in green sticks. The hydrogen bonds are labeled with yellow dotted lines. Noteworthy, the F357 in compound 5‐Lp‐PLA2 adduct (green in C) generates a −22° rotation compared with that in structure of darapladib (cyan in C), as shown by the yellow arrow points out. The figure was prepared using the program PyMOL. Lp‐PLA2, lipoprotein‐associated phospholipase A2 [Color figure can be viewed at wileyonlinelibrary.com]

Currently, FBLD has gradually matured to be a reliable and efficient approach for producing promising lead compounds for subsequent medicinal chemistry optimization, and more than 30 drug candidates derived from fragments have entered in clinical trials.^69^ On the basis of the aforementioned crystal structures, both Astex in collaboration with GSK and our group applied the FBLD strategy to identify novel Lp‐PLA2 inhibitors, consequently releasing numerous crystal structures for fragment hits or representative compounds. Typically, compounds **3** (Figure 7A), 4 (Figure 7B), and 5 (Figure 7C) displayed favorable research prospects. The crystal structure of compound 3 bound to Lp‐PLA2 reveals that the carbonyl of lactam forms two hydrogen bonds to the backbone NH of both Leu153 and Phe274, the cyano fills a small pocket by forming a long H‐bond to the backbone NH of Phe357, and the phenyl ring locates in the hydrophobic pocket aligned with Phe110, Ala355, and Phe357. Concurrently, the sulfone moiety sits in the Leu153, Trp298, and Phe322 groove, where the electron withdrawing effect of sulfone might polarize the neighboring CH that then interacts with the electron‐rich indolyl of Trp298. In particular, the linker between the phenyl and sulfone moiety is vital for maintaining the inhibitory potency, as it can orient the two parts to the respective binding pocket, and the ether oxygen forms a close contact with the side chain of Gln352. Compared with darapladib, compound **3** efficiently utilizes the groove above Trp298 to enhance the potency, as Trp298 appears to be essential for substrate interactions with Ser273. Since the groove extends toward the solvent, occupying the space can also modulate the physicochemical properties of the ligand.^34^

Very recently, our group identified compound **4** as a promising lead for further druggability studies. The crystal structure of **4** in complex with Lp‐PLA2 reveals that the phenylcarboxyl group and the diaryl ether moiety expand along the canyon‐like groove in opposite directions. The phenylcarboxyl group projects into the solvent‐accessible region close to the catalytic site and forms hydrophobic interactions with Phe110. The sulfonamide oxygen forms H‐bonding interactions with Phe274, Ser273, and Leu153, while an additional H‐bond is established between the cyano and backbone of Phe357 as in the case of compound **3**. Phe357, Leu371, Leu111, and Leu121 contribute to hydrophobic interactions via the 4‐chloro‐3‐(trifluoromethyl) phenyl moiety, which sits above the lipoprotein‐binding helices on the other side. Remarkably, Lp‐PLA2 rotates the Gln352 residue 90° from its position in the structure of the original fragment to that of compound **4**, resulting in a newly ordered conformation mediated by two H‐bonds with Lys370 and a water molecule. Nevertheless, this rotation does not seem to significantly influence the enzymatic potency or protein framework.^33^

Unlike other reversible inhibitors, compound **5** does not directly interact with the catalytic residues of the enzyme. Furthermore, the terminal tolyl moiety of the bis‐aryl group in compound **5** inserts into a new pocket and forms π‐stacking interactions with Phe125 and Phe357, resulting from a −22° rotation of the C_α_–C_β_ bond in Phe357. The rest of the tolyl is in the van der Waals contact with the side chains of Leu111 and Leu371 and forms an edge‐to‐face π‐stacking interaction with Phe357. Additionally, two direct hydrogen bonds can be found between the amino of the aminoethyoxy moiety and the Leu369 carbonyl as well as between the hydroxyl of the hydroxylmethy moiety and the Gln352 amino. A small lipophilic subpocket formed by the side chains of Ala355, Tyr160, and Phe357 is appropriately filled by the methoxy moiety. In addition, a number of water‐mediated contacts with a network of waters are found across the top of the pocket, which may also contribute to improving the affinity.^35^

The structural information obtained from the complexes between Lp‐PLA2 and several reversible inhibitors validate that the inhibitor‐binding pocket is fairly open and large and those hydrophobic interactions play a major role in binding. Except for compound **5**, the other five compounds share common pharmacophore features, such as a carbonyl or sulfonyl occupying the oxyanion hole, a hydrophobic group located in the lipophilic pocket above two solvent‐accessible α‐helices, and a hydrophilic moiety extending toward the solvent area. Compound **5** is still effective in inhibiting Lp‐PLA2 though it does not occupy the active site, further suggesting a larger inhibitor‐binding pocket. Moreover, specific interactions with Trp298 or its adjacent residues might contribute to the improved potency as compound **3** did. Altogether, the elucidation of the binding mechanism and detailed interactions of the reversible inhibitors with Lp‐PLA2 provide valuable information for further design and development of novel Lp‐PLA2 inhibitors. More importantly, the successful application of FBLD in identifying novel efficient Lp‐PLA2 inhibitors with vastly improved physicochemical properties can be used for reference in other targets.

## 3 GENETIC VARIABILITY AND REGULATION OF EXPRESSION

The gene encoding Lp‐PLA2 (*PLA2G7*), whose chromosomal location is in 6p12‐21.1, is organized in 12 exons and encodes 441 amino acids.^70^, ^71^ Numerous variations were identified within *PLA2G7*, and the corresponding alterations in physiology and body homeostasis have been illustrated to varying degrees. Since elevated Lp‐PLA2 levels were detected in patients with certain diseases, most researchers have focused their attention on variants that considerably weaken Lp‐PLA2 activity or function, viz the loss‐of‐function alleles. The functional and clinical consequences associated with *PLA2G7* polymorphisms were summarized in a review by Stafforini^24^ in 2009, which mainly discussed the research status of Val279Phe, Arg92His, Ile198Thr, and Vla379Ala SNPs at that time. The clinical consequences were largely focused on cardiocerebrovascular diseases, which was considered the most promising field in which Lp‐PLA2 was involved. In addition, several diseases that were rarely reported to be associated with *PLA2G7* polymorphisms, such as asthma, schizophrenia, CKD, multiple sclerosis, ulcerative colitis, and Kawasaki disease. Notably, however, the results of most research mentioned in that review, regardless of functional or clinical consequences, were inconsistent.^24^ In this review, we also described the research state of the *PLA2G7* polymorphisms from two aspects, the functional and clinical consequences, while emphasis will be placed on recent results.

### 3.1 Val279Phe mutation (rs76863441)

A base G to T transition at nucleotide position 994 in exon 9 of *PLA2G7* leads to a Val to Phe substitution at residue 279, which is in proximity to the active site of the enzyme. The V279F mutant gene is codominantly inherited, and the mutant protein is almost undetectable in the plasma of a homozygous person (279FF), as the misfolded protein cannot be secreted normally.^24^ The prevalence of 279FF was reported to be 0.4%, 1.2%, and 3% in the Chinese, Korean, and Japanese populations, respectively, while it is rare in the Middle Eastern population and almost completely absent in Europeans.^72^ On the basis of the regional nature of the prevalence, the correlations between the V279F mutation and diseases was widely determined in the East Asian population and focused on the cardiocerebrovascular field.

The clinical consequences associated with the V279F polymorphism were summarized in separate reports by Stafforini^24^ and Karasawa,^73^ in which almost all the results were obtained before 2010. However, in contrast to the distinct effect on Lp‐PLA2 activity, the influence of the V279F mutation on specific diseases remains unclear. The results from the studies discussed in these two reviews were inconsistent, as positive, negative, and nonsignificant associations between V279F mutation and diseases were all described in different studies, even for the same type of disease. Similarly, such contradictory results were observed in several literature reports over the past couple of years (Table 1). Earlier, two meta‐analyses aiming to determine the association between the V279F polymorphism and coronary heart disease (CHD) risk did not support a role of V279F in increased risk of CHD (the F allele vs the V allele: odds ratio (OR) = 1.14, 95% confidence interval (CI): 0.86‐1.52; OR = 1.09, 95% CI: 0.88‐1.35, respectively).^74^, ^75^ This observation was further substantiated by two recent large‐scale human genetic studies. One was in a Chinese population, and almost 90 000 individuals with the F279 allele^76^ were randomly selected for identification the associations of V279F with various vascular diseases. However, neither positive nor negative correlations were demonstrated between V279F and any vascular diseases. Another large correlative study also illustrated that the V279F polymorphism was not related to CHD risk (OR = 0.95, 95% CI: 0.88‐1.03).^77^ Nonetheless, harboring the F279 allele was identified as protective against the development of CAD in South Korean males (OR = 0.80, 95% CI: 0.66‐0.97, *P* = .02).^78^ Interestingly, inconsistent results were obtained even for the same ethnic population and disease as well. For example, a case‐control study demonstrated no significant association between the V279F mutation and ischemic stroke in a Chinese population (OR = 0.82, 95% CI: 0.44‐1.24),^79^ whereas another genetic study established an increased risk despite using the same study population and disease (OR = 2.51, 95% CI: 1.02‐5.91, *P* = .037).^80^ In the case of atherosclerosis, a significant inverse association of V279F polymorphism with clinical atherosclerosis was indicated in a recent meta‐analysis (OR = 0.88, 95% CI: 0.81‐0.95, *P* = .0007),^81^ whereas another study did not reveal any significant associations between the V279F genotype and subclinical atherosclerosis using Mendelian randomization analyses.^82^ Therefore, the dramatic reduction of Lp‐PLA2 levels in plasma resulting from the V279F mutation is apparent, but the relationship between the V279F mutation and cardiocerebrovascular diseases is unclear. In this context, deeper and more comprehensive research may be necessary to elucidate the actual role of the V279F mutation in the pathology of cardiocerebrovascular diseases.

**Table 1:** Summary of selected studies of V279F variant recently

Study (year)	Population	Clinical status	*n*	Minor allele frequency, %	*P*
Jang et al (2011)^78^	Korean man	Controls	3128	12.8	
		CVD	2890	11.5	.02
Millwood et al (2016)^76^	Chinese	Controls	81 489	N/A	
		Major coronary events	922		N/S
		All stroke	5967		N/S
		Major vascular events	7141		N/S
Gregson et al (2017)^77^	European and South Asian ancestry	Controls	110 209	0.05	
		CHD	72 655	0.04	N/S
Chi et al (2018)^104^	Chinese	Controls	638	23.4	
		CHD	631	28.1	N/S
Hong et al (2015)^103^	Southern Chinese	Controls	414	3.9	
		CHD	322	3.4	N/S
Ni et al (2017)^80^	Chinese	Controls	260	2.3	
		Ischemic stroke	348	5.2	.037
Ma (2017)^79^	Chinese	Controls	740	5.3	
		Ischemic stroke	750	5.2	N/S
Ma et al (2017)^85^	Chinese	Controls	98	7.4	
		Acute pancreatitis	94	2.1	.014
Koshy et al (2010)^86^	Japanese	Controls	2079	16.2	N/S
		Alzheimer's disease	1952	16.0	
Fan et al (2010)^83^	Chinese Han woman	Controls	315	3.0	
		Polycystic ovary syndrome	346	6.9	.010
Zhang et al (2017)^84^	Chinese Han woman	Controls	501	2.0	
		Polycystic ovary syndrome	565	3.7	.020
Zheng et al (2017)^93^	Chinese Han	Controls	201	9.7	
		CHD with no BSS	171	6.1	N/S
		CHD with BSS	212	6.8	N/S

*Note*: The “*n*” column represents the number of participants in each study category and includes subjects harboring 0‐1‐2 polymorphic alleles. The “*P*” column represents whether the polymorphism incidence is statistically significant differences between control and diseased populations, where available/appropriate.

Abbreviations: BSS, blood stasis syndrome; CHD, coronary heart disease; CVD, cerebrovascular disease; N/A, not applicable; N/S, not statistically significant.

Additionally, the potential contribution of the V279F mutation to the occurrence or progression of other diseases was tested in recent studies. For example, both genotype and allele frequencies of the V279F mutation in Chinese populations with polycystic ovary syndrome (PCOS)^83^, ^84^ and acute pancreatitis (AP)^85^ were significantly higher than those in controls. Furthermore, multifactor logistic regression analysis revealed that the V279F mutation was an independent risk factor for AP (OR = 4.03, 95% CI: 1.27‐12.7, *P* = .018). However, the V279F mutation was not associated with a reduced risk of AD in a Japanese population (OR = 0.98, 95% CI: 0.86‐1.12, *P* = .81).^86^ Interestingly, a recent study demonstrated that overweight Korean individuals harboring the F allele exhibited lower systolic and diastolic blood pressure than those harboring the V allele (both *P* < .05) at the end of the 3‐year follow‐up, though no significant differences were found at baseline. This result implied that the F allele might protect carriers against hypertension, even in the case of persistent overweight for longer than 3 years.^87^ Nevertheless, some potential limitations existed in these studies, such as lower power in subsidiary analyses due to small sample size and incomplete conclusions regarding the effect of the V279F mutation on diseases resulting from specific inclusion criteria.

### 3.2 Val379Ala mutation (rs1051931)

A systematic screening of the *PLA2G7* gene revealed a base A to G alteration at position 1136 of exon 11, which resulted in a valine to alanine replacement at residue 379.^88^ Peculiarly, the ancestral allele at this position is base A, but the frequency of mutant base G predominantly reaches approximately 80%.^77^ Although the V379A mutation is relatively common in European and African populations, its effect on plasma Lp‐PLA2 activity is unclear until recently. Earlier, Casas et al^89^ and Grallert et al^90^ performed two large meta‐analyses in individuals of European descent and found a significant additive increase in Lp‐PLA2 activity resulting from the V379 allele. In 2014, Chae et al^91^ identified that the V379 allele was associated with increased Lp‐PLA2 activity in Koreans as well. The increase of plasma Lp‐PLA2 activity in the above studies might derive from the wild‐type V379 allele though only to a small extent (<10%), that is, a moderate decrease in plasma Lp‐PLA2 activity could be attributed to the mutant A379 allele. Indeed, a recent large‐scale study found that Lp‐PLA2 activity could be decreased by 2.7% for each A379 allele inherited (percentage mean difference: −2.74%; 95% CI: −3.51 to –1.98).^77^ On the other hand, several studies reported that no significant impact of the V379 allele was observed on Lp‐PLA2 activity. Qi et al^92^ revealed no statistically significant effect of the V379A polymorphisms on Lp‐PLA2 activity in a Chinese Han population (*P* > .05). A series of studies on *PLA2G7* genetic polymorphisms reported similar conclusions in Chinese subjects with PCOS (*P* > .05),^84^ AD (*P* = .33),^85^ and CHD with (*P* = .21) or without blood stasis syndrome (BSS) (*P* = .98).^93^ Interestingly, two studies demonstrated increased Lp‐PLA2 activity in A379 allele carriers. The relatively small‐scale study described dramatically increased activity in homozygous AA subjects (28.9 ± 6.9 nmol/mL) compared with homozygous VV subjects (5.6 ± 2.3 nmol/mL, *P* < .001), while the large one detected only a moderate increase.^38^, ^94^ However, functional studies revealed that the Val379 would lower the substrate affinity (namely, higher Michaelis‐Menten constant, *K*_M_) and increase the maximal velocity (*V*_max_) in vitro.^95^ Hence, the effect of the V379A mutation on plasma Lp‐PLA2 activity is controversial thus far, but the degree of action may be limited for each direction.

Due to its potential impact on plasma Lp‐PLA2 activity, the V379A mutation was explored in patients with a variety of diseases. Compared with the ancestral allele A (V379 allele), the derived allele G (A379 allele) presents at a much higher frequency. Therefore, most studies have concentrated on the influence of the minor V379 allele on diseases, in which this allele was regarded as the mutant variant. In the Chinese population, the frequencies of the A379 allele were not significantly different between patients with AP (*P* = .71) or PCOS (*P* = .88) and their respective healthy controls. Moreover, no associations were observed between the V379A genotype and the risks of AP or PCOS (both *P* > .05).^84^, ^85^ As in the case of V279F polymorphism, the correlation studies largely focused on the associations between V379A mutation and vascular diseases. However, since Stafforini's review, only one investigation has demonstrated a significant association of the V379A polymorphism with vascular diseases. Liu et al^96^ detected a role of the V379A variant in increasing the risk of ischemic stroke in northern Chinese Han patients (OR = 1.43, 95% CI: 1.02‐2.00, *P* = .04). Due to the significant association of the heterozygous (VA) genotype with large‐artery atherosclerotic stroke, even after adjusting for confounding factors (OR = 1.67, 95% CI: 1.13‐2.48, *P* = .01), the authors concluded that the V379A variant might contribute to ischemic stroke susceptibility in the northern Chinese Han population.^96^

The majority of the newly reported studies observed no obvious contribution of the V379A variant to the susceptibility and severity of vascular diseases. Two studies involving more than 500 participants demonstrated that the V379A polymorphism was not significantly linked with the risk of CHD; one of these studies was in a Chinese population (OR = 1.25, 95% CI: 0.78‐1.99),^93^ while the other was in a white Italian population (*P* > .05).^97^ Similarly, a large‐scale genetic study deliberated the relationship of the V379A variant with CHD and angiographic CAD, but no apparent association was observed (OR = 0.96, 95% CI: 0.89‐1.03; OR = 0.96, 95% CI: 0.87‐1.06, respectively).^89^ Akin to this conclusion, another recent study with a large enough sample (82 907 CHD patients in the case cohort and 147 029 healthy controls) determined that the rare V379 allele was not related to CHD risk (OR = 1.01, 95% CI: 0.68‐1.51).^77^ Furthermore, no difference in the effects of the V379 and the A379 alleles on CHD was reported in two meta‐analyses, one in 2010 by Wang et al^74^ (OR = 0.99, 95% CI: 0.85‐1.15, *P* = .89) and the other in 2017 by Santoso et al^81^ (OR = 1.08, 95% CI: 0.93‐1.26, *P* = .31). In summary, although the relationship between the Lp‐PLA2 level and CHD risk was inconclusive, the V379A variant is unlikely to be an apparent CHD risk.

### 3.3 Arg92His mutation (rs1805017)

In addition to the V379A mutation, Bell et al^88^ discovered that the Arg92His mutation, an arginine to histidine transition at codon 92, is a common polymorphism in Caucasian subjects. Moreover, the impact of this polymorphic variant on plasma Lp‐PLA2 level was as inconsistent as that of the V379A mutation. A functional study by Kruse et al^95^ demonstrated comparable kinetic characteristics between the recombinant mutant Lp‐PLA2 and wild type with respect to *K*_M_ and *V*_max_. Similarly, Qi et al^92^ found that R92H was not associated with Lp‐PLA2 mass or activity in the Chinese Han population, even after Bonferroni correction. Nevertheless, a genome‐wide association study including data from ﬁve community‐based studies revealed a strong association of the R92H mutation with increased Lp‐PLA2 mass (*P* = 2.4 × 10^−23^, significance threshold *P* < 5 × 10^−8^), but a relatively weak association with Lp‐PLA2 activity (*P* = 2.4 × 10^−6^).^90^ Furthermore, three studies successively discerned that a significantly increased Lp‐PLA2 level was associated with the H92 allele in the Chinese population, and of these, two were about Lp‐PLA2 mass and the third was about activity.^84^, ^85^, ^93^ Interestingly, Suchindran et al^98^ established that the H92 allele was significantly associated with increased plasma Lp‐PLA2 mass (*P* = 5.8 × 10^‐14^) and diminished Lp‐PLA2 activity (*P* = 1.5 × 10^−3^, significance threshold *P* < .01). Similarly, Maiolino et al^97^ also observed the opposite effects on Lp‐PLA2 mass and activity in high‐risk CAD patients of European ancestry. In addition, a large genetic study revealed a trend toward an inverse association between Lp‐PLA2 activity and the H92 allele (genotype HH vs RR, OR = −0.02, 95% CI: −0.05 to 0.00).^89^ In a sizable pharmacogenetic analysis reported recently, Yeo et al^38^ found that the R92H common variant was associated with a slightly reduced Lp‐PLA2 activity. As the authors of the aforementioned three studies provided only data on Lp‐PLA2 activity, we could not compare the effect on Lp‐PLA2 mass among them.^38^ Though divergent, the impact of the R92H polymorphism on plasma Lp‐PLA2 levels might be modest, and the characteristic biochemical properties of the protein would not be significantly altered.^99^

Comprehensive genetic analyses were carried out to determine the influence of the R92H variant on the susceptibility and severity of various diseases. Similar to the previous two variants, researchers also explored the associations of the R92H polymorphism with AP^85^ and PCOS.^84^ Consequently, a significant association was observed only in AP patients (OR = 1.96, 95% CI: 1.02‐3.76, *P* = .043), in whom the frequency of the H92 allele was higher than that in the healthy controls.^85^ For vascular diseases, the effect of the R92H variant was inconsistent in different studies. Along the lines of the results summarized by Stafforini in the European population,^100^, ^101^ the H92 variant was associated with an increased risk of vascular diseases in the Chinese population by Zheng et al^93^ (OR = 1.81, 95% CI: 1.18‐2.78),^93^ Xu et al^102^ (OR = 1.63, 95% CI: 1.02‐2.62), Ma^79^ (OR = 1.42, 95% CI: 1.02‐2.00), Hong et al^103^ (OR = 2.66, 95% CI: 1.50‐4.69), and Chi et al^104^ (OR = 1.45, 95% CI: 1.16‐1.92). Additionally, a recent meta‐analysis also yielded a significantly positive association between R92H mutation and clinical atherosclerosis (OR = 1.29, 95% CI: 1.09‐1.53).^81^ However, Casas et al^89^ (OR = 1.07, 95% CI: 0.84‐1.36) reported a nonsignificant impact of H92 allele on the risk of CHD in a large‐scale genetic study. Nevertheless, only one study, as far as we know, demonstrated a protective role of the H92 variant in acute myocardial infarction (AMI) patients, as patients harboring the wild‐type genotype (RR) exhibited poorer AMI‐free survival than patients with the RH and HH genotypes during an 8‐year follow‐up (*P* = .003).^97^

### 3.4 Ile198Thr mutation (rs1805018)

The I198T mutation, first termed the Iso195Thr polymorphism, was another common frequency variant found by Bell et al^88^ in 1997. The impact of the I198T variant on the plasma Lp‐PLA2 level remained inconclusive.^88^ Qi et al^92^ found an evident reduction in both Lp‐PLA2 mass and activity when carriers homozygous for the minor allele (TT) were compared with the major allele homozygotes (II) in a Chinese population.^92^ Maiolino et al^97^ reported increased Lp‐PLA2 activity and unnoticeably altered Lp‐PLA2 mass in participants harboring the T198 allele. Interestingly, no significant effect of the I198T variant was observed on either the Lp‐PLA2 mass or activity in a translational study.^105^ Similarly, Zheng et al^93^ also obtained a comparable noncorrelation between the I198T variant and Lp‐PLA2 mass. In addition to in vivo studies, Kruse et al^95^ conducted kinetic experiments with the recombinant enzyme containing the I198T variant, revealing a sixfold higher *K*_M_ value and comparable *V*_max_ than the wide‐type enzyme. Consequently, the authors speculated that the variant influences the plasmatic Lp‐PLA2 protein by lowering the substrate affinities rather than its hydrolytic activity.

Since its discovery together with the V379A and R92H mutations more than 20 years ago, a number of studies have been carried out to clarify the involvement of the I198T variant in a variety of diseases. A recent study revealed that T198 allele showed no significant effect on aging in Sicilian octogenarians and either risk or age of onset of AD in a Japanese population.^86^ Similarly, Sankararaman et al^106^ reported no association between the haplotype of V379A and I198T genotypes and necrotizing enterocolitis in infants in northwest Louisiana, and the variant frequency was not significantly different from that of healthy controls (*P* = .26). However, beyond these reports, studies to determine the role of the I198T variant in diseases other than vascular abnormities have rarely been reported in the past few years. Similar to other polymorphisms in *PLA2G7*, the results of the correlation research on this allele in vascular disease are still complicated. Hong et al^103^ found that the T198 allele would not affect the risk of CHD in a Chinese population (OR = 1.26, 95% CI: 0.85‐1.87). Likewise, Ferguson et al^105^ failed to uncover a significant association between the I198T polymorphism and coronary artery calcification (CAC). A recent meta‐analysis also revealed no significant association between the T198 variant and atherosclerosis (OR = 1.12, 95% CI: 0.79‐1.59), whereas weak statistical significance was obtained in a subgroup analysis of the Asian/Oriental population (OR = 1.26, 95% CI: 1.06‐1.51).^81^ Furthermore, Zheng et al^93^ demonstrated that the T198 allele was strongly associated with the risk of CHD with BSS (OR = 1.93, 95% CI: 1.21‐3.06) but not associated with the risk of CHD without BSS (OR = 1.47, 95% CI: 0.89‐2.44). Such a limited association was also observed in three studies included in Stafforini's review.^101^, ^107^, ^108^ Interestingly, two additional unrelated studies revealed quite different effects of the T198 allele on cardiovascular disease (CVD). Both in SNP (OR = 0.69, 95% CI: 0.53‐0.90) and haplotype analyses, Hoffmann et al^109^ reported lower risks of CAD resulting from the T198 allele, which was more frequent in healthy controls. However, this mutation was not significantly associated with the survival outcome in the subsequent analysis regarding all‐cause and cardiovascular mortality. In contrast, Chi et al^104^ identified that the T198 allele was more prevalent in CHD patients and was associated with a higher risk of CHD (OR = 1.51, 95% CI: 1.23‐1.97). Although these studies were carried out in different populations, the clearly opposing results highlight the need for further investigations to elucidate whether this genetic variant is associated with the risk and outcome of CVD.

### 3.5 Other SNPs

A number of other missense mutations were explored in relation to their effect on either plasma Lp‐PLA2 level or disease progression but were usually of lower prevalence in the studied population. The Gln281Arg mutation (rs201256712) could lead to a Gln to Arg replacement at residue 281, which occurs rarely in exon 9 of *PLA2G7* (minor allele frequency (MAF) < 0.5%).^110^ Three studies determined the impact of the variant on plasma Lp‐PLA2 activity and revealed its effect on the reduction in enzymatic activity, though to a different extent among the three investigations.^77^, ^110^, ^111^ Furthermore, two studies using computational modeling found that the Q281R variant was deleterious to the structure and function of Lp‐PLA2.^112^, ^113^ To identify rare loss‐of‐function mutations of the Lp‐PLA2 gene (MAF < 0.5%), Song et al^99^ resequenced the exons of *PLA2G7* in 2000 samples from a Western European population. Among the 19 nonsynonymous SNPs obtained, the Arg82His mutation (rs144983904, MAF = 0.08%) was predicted to be damaging for protein function.^99^ Two large‐scale recent genetic studies also reported a similar negative impact on plasma Lp‐PLA2 activity, while all three investigations failed to find an association of this mutation with various cardiovascular traits.^38^, ^77^ The I317N mutation (rs201842579) was first demonstrated in Japanese subjects who carried an A to T transition at nucleotide 950 in exon 10 and a substitution of Asn for Ile at codon 317.^111^ Such a rare mutation was likely to generate a new N‐linked glycosylation site on Lp‐PLA2, disturb the secretion of the mature enzyme, and result in a deficiency in plasma Lp‐PLA2 levels. This finding was supported by Yeo et al^38^ in a large pharmacogenetic meta‐analysis, where they described decreased enzymatic activity resulting from the I317N mutation. In addition, the authors revealed that the rare Leu389Ser variant (rs34159425, MAF = 0.03%) was deleterious to Lp‐PLA2 activity and that the rare Thr278Met variant (rs147252565, MAF = 0.01%) was also likely to be a null allele but was too uncommon to generate significant results.^38^ Similarly, Polfus et al^114^ found that the L389S mutation was strongly associated with decreased Lp‐PLA2 activity but not with incident CHD (hazard ratio [HR] = 0.92, 95% CI: 0.35‐1.49, *P* = .78) or cardiovascular‐related mortality in African‐Americans.

In addition, several research groups have tried to detect the consequences of SNPs other than the nonsynonymous variants within the *PLA2G7* gene region. A nonsense variant at codon 287, Gln287Ter or Q287X mutation (rs140020965), resulted in premature termination of protein translation. In all three studies mentioning this rare variant, researchers found that carriers had greatly reduced plasma Lp‐PLA2 activity but no significant difference in the risk of CHD compared with noncarriers.^38^, ^77^, ^114^ Another premature termination was observed at codon 63 due to an insertion of adenine at nucleotide 191 in exon 3 (Ins191), which would partly impair enzymatic function to a degree.^111^ Intron mutations were also assessed for their contribution to plasma Lp‐PLA2 level or disease progression. Among them, rs7756935 caught the attention of researchers, probably owing to the perfect linkage disequilibrium with the Val379Ala mutation (rs1051931). Like the V379A variant, the ancestral allele C of rs7756935 is the minor frequency allele and is commonly prevalent in the population (MAF is approximately 19%). Grallert et al^90^ reported that the minor allele of rs7756935 was associated with an increased Lp‐PLA2 activity but not with Lp‐PLA2 mass, while no significant effect was observed by Ferguson et al^105^ on either mass or activity. Further, neither demonstrated an association between the rs7756935 and CHD or CAD. On the other hand, Xu et al^102^ revealed that the minor allele C of rs7756935 acted as a protective factor against CHD in Chinese females (OR = 0.59, 95% CI: 0.35‐1.00, *P* = .05). Rs2216465 is another well‐studied mutation, in which the rare allele homozygotes might have lower plasma Lp‐PLA2 activity than the common allele homozygotes.^89^ Casas et al^89^ revealed that this intronic variant was not associated with CHD, and Sutton et al^101^ detected only a nominally significant association between rs2216465 and CAD outcome in their study, where the significance disappeared after multifactor adjustment. However, Ferguson et al^105^ did not observe an association between rs2216465 and plasma Lp‐PLA2 mass or activity, whereas a significant association was detected with CAC. In addition, a number of other intronic variants in the *PLA2G7* gene were also explored, and their impact on the function and expression of Lp‐PLA2 was preliminarily demonstrated with respect to the susceptibility and severity of diseases.^90^, ^105^

Furthermore, numerous variants located in the 5′ upstream, 5′‐untranslated region (5′‐UTR), and 3′‐UTR might be associated with the plasma Lp‐PLA2 level and even certain diseases. Rs1421378, an intergenic variant, might impair plasma Lp‐PLA2 activity with a gene‐dose effect,^89^ although two studies did not observe any significant association.^98^, ^105^ Moreover, Casas et al^89^ were unsuccessful in detecting the clear involvement of this common variant with CHD risk, while Ferguson et al^105^ demonstrated a strong association of rs1421378 and CAC. A 5′‐UTR variant (rs9395208) might not be associated with Lp‐PLA2 activity and mass in the Chinese population,^92^ but in the same population, another study found the minor allele of rs9395208 to be a protective factor for CHD (OR = 0.78, 95% CI: 0.62‐0.98, *P* = .03).^115^ However, Grallert et al^90^ established that this variant was significantly associated with Lp‐PLA2 mass but not with activity or prevalence of CHD or CAD in participants of European ancestry. Rs12528857 is an example of a 3′‐UTR variant that might increase plasma Lp‐PLA2 mass without affecting the activity.^98^ In this context, Sutton et al^101^ did not observe any association between this variant and CAD outcomes in tested subgroups after adjusting for cardiovascular risk factors. In addition, Gregson et al^77^ regarded a splice donor variant, c.109 + 2T > C (rs142974898), as a loss‐of‐function mutation that was unrelated to CHD risk. Interestingly, Jiang et al^116^ found that the elevated methylation of *PLA2G7* promoter might increase the risk of CHD in Chinese females; however, only 36 CHD patients and 36 age‐ and sex‐matched controls were investigated.^116^

Over the past 20 years, substantial efforts have been made to study Lp‐PLA2 at the genetic level. Two heritability studies independently reported that genetic factors might account for 54% and 62% of the variation in Lp‐PLA2 activity,^117^, ^118^ while another larger study attributed only 37% to genetic variance.^119^ In addition, numerous variants throughout the *PLA2G7* gene were investigated to determine their functional and clinical outcomes. Certain variants are more prevalent in specific populations, such as the V279F and I317N variants in East Asians, the Q287X variant in whites and the L389S variant in African ancestral populations. By contrast, the minor alleles of the V379A, R92H, and I198T variants are more common among various ethnic groups, with a frequency of more than 5%. With respect to the impact of these variants on the plasma Lp‐PLA2 level, seemingly only the V279F mutation was uniformly agreed to be deleterious, whereas the functional consequences of other variants were inconsistent among different investigations. Thus, in future genetic analyses of human diseases, multivariant loci might be a good choice as the instrumental variable of Mendelian randomization, as the *PLA2G7* gene presently lacks a representative SNP with common frequency in diverse ethnic groups and a verified effect on Lp‐PLA2. In addition, the variants in the *PLA2G7* gene, which are likely to affect gene expression or interactions with lipoproteins, may also result in varying plasma Lp‐PLA2 levels.

Usually, a significant association between elevated plasma Lp‐PLA2 activity and increased risk of CHD could be detected, and certain SNPs were also found to be responsible for the altered activity. However, a number of large genetic studies failed to demonstrate any association between SNPs and CHD risk, while several other reports described either a positive or a negative association. In particular, in a recent large‐scale study that included more than 260 000 total participants, the researchers genotyped four rare loss‐of‐function mutations (c.109 + 2T > C, R82H, V279F, and Q287X) and one common modest‐impact variant (V379A) in *PLA2G7*. They compared the effects of darapladib treatment and Lp‐PLA2‐lowering alleles on plasma Lp‐PLA2 activity, conventional cardiovascular risk factors, and CHD risk. As a result, none of the five functional alleles, which induced a widely differing degree of reduction in Lp‐PLA2 activity, was connected to CHD risk. Thus, the authors concluded that Lp‐PLA2 is unlikely to be a causal risk factor in CHD.^77^ In contrast, Ferguson et al^105^ found a nonsignificant association of numerous SNPs in *PLA2G7* with Lp‐PLA2 activity or mass, but a strong association with CAC. Consequently, they concluded that genetic variation in *PLA2G7* might relate to CHD independent of the circulating Lp‐PLA2 levels. Although the clinical consequence of *PLA2G7* SNPs for vascular complications, particularly macrovascular abnormalities, remains unclear, they do not seem to be either risk or protective factors. Furthermore, *PLA2G7* polymorphisms were explored to verify their effects on subjects receiving therapy with certain drugs. During acetylsalicylic acid therapy, Postula et al^120^ and Peng et al^121^ observed that the minor allele of rs7756935 was associated with increased platelet activity in Caucasians with type 2 diabetes and a Chinese population with ischemic stroke, respectively, signifying a higher risk of aspirin resistance in carriers than noncarriers. In addition, the I198T variant might be responsible for the interaction effects between genotype and omega‐3 polyunsaturated fatty acid (n‐3 PUFA) supplementation on plasma triglyceride (TG) levels.^122^ However, a recent pharmacogenetic study revealed that none of the variants associated with Lp‐PLA2 activity, including the *PLA2G7* gene, was related to any efficacy endpoints of darapladib treatment.^38^ In summary, although much progress has been made in this field, the inconsistent results require further comprehensive and powerful research to eliminate divergence. Moreover, the knowledge acquired from the studies on vascular diseases could be applied to other diseases, which might uncover the unanticipated impact of *PLA2G7* variants on certain diseases or specific treatments.

### 3.6 Transcriptional regulation of Lp‐PLA2 expression

Hematopoietic stem cell‐derived cells, such as monocytes, macrophages, mast cells, and T‐lymphocytes, are the main sources of the Lp‐PLA2 protein.^25^, ^123^ Messenger RNA (mRNA) for Lp‐PLA2 is extensively detected in several kinds of tissues, which is most likely the result of the expression of macrophages in these tissues.^123^ Analogous to the housekeeping genes, the *PLA2G7* promoter lacks a TATA box but harbors a set of GC‐rich motifs adjacent to the transcription start site; however, it seems more like a tissue‐specific gene that has the same TATA‐less promoter.^11^ Stafforini et al determined the transcriptional initiation site and identified the essential elements of the promoter region that afforded maximal basal promoter activity. Additionally, they observed negative regulation when reporter constructs consisted of specific additional 5′ upstream sequences.^123^ A further cellular and biochemical investigation regarding human macrophages demonstrated that the transcription factors Sp1 and Sp3 bound to noncanonical Sp‐binding sites localized within the minimal promoter regions of the human *PLA2G7* gene, which was vital for basal Lp‐PLA2 expression. Therefore, the expression of the *PLA2G7* gene closely involves members of the Sp family of transcription factors and finely tuned modulation mechanisms.^124^

### 3.7 *PLA2G7* gene regulation and cellular differentiation

Initially, a number of research groups identified a dramatic upregulation of Lp‐PLA2 expression during macrophage differentiation.^25^ The presence of multiple potential binding sites for MS2 and other differentiation‐induced transcription factors in the promoter region of *PLA2G7* indicated that Lp‐PLA2 expression might be under tight differentiation control. A subsequent study reported higher levels of Sp1/Sp3 binding activity in macrophages than in monocytes. Along with the expression profile of Sp1, which is at a high level in the Lp‐PLA2 expression tissues, these observations might account for the elevated gene expression level during the maturation of monocytes to macrophages and revealed the participation of Sp1/Sp3 in this process.^124^ Ferguson et al^105^ supported this differential expression in the process of macrophage maturation and found that Lp‐PLA2 expression continuously increased during further polarization to M1 macrophages and foam cells but not to M2 macrophages (anti‐inflammatory phenotype). Therefore, the fluctuating Lp‐PLA2 levels among different states of cellular differentiation probably resulted from the inflammatory response.^105^

Apart from macrophages, *PLA2G7* also seems to play a crucial role in the differentiation of SMCs and megakaryocytes. Xiao et al^125^ observed that *PLA2G7* gene expression was significantly increased during SMC differentiation from stem cells. A further study revealed that enforced expression of *PLA2G7* significantly promoted the binding of serum response factor to SMC differentiation gene promoters, resulting in SMC differentiation.^125^ Similarly, increased expression of Lp‐PLA2 mRNA and protein occurred during the course of CD34^+^ cell differentiation toward megakaryocytes. Certain lipid mediators, which accumulate when the endogenous Lp‐PLA2 activity was neutralized in megakaryocytes, could activate the PAF receptor to control the megakaryocyte α_IIb_β_3_‐dependent adhesion, cell spreading, and mobility. These observations indicated that the increased expression of Lp‐PLA2 during megakaryopoiesis might shield the function and development of megakaryocytes from damage by detrimental intracellular phospholipid mediators.^126^

### 3.8 Regulation of Lp‐PLA2 expression in response to exogenous stimuli

At present, numerous exogenous stimuli have been reported to regulate the secretion of Lp‐PLA2, such as substrates, inflammatory or anti‐inflammatory agents, and a range of cytokines and steroid hormones. In vitro, a number of cytokines, such as interleukin 1α (IL‐1α), IL‐1, IL‐4, IL‐6, tumor necrosis factor‐α (TNF‐α), interferon‐γ (IFN‐γ), IFN‐α, granulocyte‐macrophage colony stimulating factor, and macrophage colony stimulating factor (M‐CSF), could decrease Lp‐PLA2 secretion from human macrophages to varying degrees. However, stimulation with IL‐1β, G‐CSF, and TNF‐α might amplify the secretion of functional Lp‐PLA2 from less differentiated cells, signifying that the state of cellular differentiation was also able to affect the responses to exogenous stimulation.^123^, ^127^ Additionally, enzymatic substrates, such as PAF and oxidized low‐density lipoprotein, were also identified to upregulate Lp‐PLA2 expression at the cellular level.^25^, ^41^, ^128^, ^129^ In vivo, Miyaura et al^130^ found that dexamethasone and progestins could augment the Lp‐PLA2 levels in the plasma of adult male and female rats, but estrogens produced a completely opposite effect.^130^

Lipopolysaccharide (LPS), a component of cell walls of Gram‐negative bacteria, might be principally responsible for the inflammatory reaction elicited by the bacteria. LPS stimulation affects Lp‐PLA2 expression both in vitro and in vivo, but the results were contradictory. Early studies in cellular models revealed negative regulation by LPS and inhibition of the secretory process of human decidual macrophages,^131^ 12‐*O*‐tetradecanoyl phorbol‐13‐acetate (TPA)‐differentiated HL (human promyelocytic leukemia cell line)‐60 cells^127^ and cultured Kupffer cells.^39^ However, a subsequent publication reported an increase in Lp‐PLA2 expression with LPS challenge to both the murine macrophage cell line RAW264.7 and human THP‐1 cells that overexpressed CD14.^132^ Furthermore, the authors described that the p38 mitogen‐activated protein kinase (MAPK) pathway participated in the LPS‐mediated modulation of *PLA2G7* expression and further determined an enhanced transactivation mediated by Sp1.^132^ In human nonadherent monocyte/macrophage cells (Mono/Mac 6; MM6), Howard et al^133^ also found that LPS induced Lp‐PLA2 expression in a p38 MAPK‐dependent manner. The ability of LPS to alter Lp‐PLA2 expression was explored in diverse tissues from a variety of animal models. Howard et al^134^ detected augmented expression of Lp‐PLA2 in several tissues at 24 hours after systemic administration of LPS to male Sprague‐Dawley (SD) rats, where the greatest increase was detected in the lung followed by the spleen, liver, kidney, and thymus. In addition, a twofold increase in plasma Lp‐PLA2 activity was detected at 24 hours following LPS exposure, but no change was observed at 12 hours. In male SD rats, Svetlov et al^135^ described a maximal four‐ to fivefold increase in bile Lp‐PLA2‐specific activity at approximately 2.5 hours after LPS infusion, followed by a gradual decline to baseline levels within 18 hours. However, plasma Lp‐PLA2 activity was significantly elevated 18 hours after LPS exposure, whereas no difference was detected at 5 hours compared with saline injection.^135^ In Syrian hamsters, the liver, spleen, lung, and small intestine exhibited strikingly increased Lp‐PLA2 mRNA levels after a 16 hours exposure to LPS, and the increase in plasma peaked at 24 hours and was sustained for at least 48 hours.^136^ The increased plasma Lp‐PLA2 activity was also determined in two other studies after LPS administration in C57BL/6 mice.^136^, ^137^ Nevertheless, when a lethal dose of LPS was injected into Swiss mice, plasma Lp‐PLA2 activity was found to decrease as observed in sepsis patients.^138^ Similarly, two studies reported a diminished plasma Lp‐PLA2 activity in response to LPS challenge in a sepsis mouse model,^137^ and one of them resulted in a noticeable decline that persisted for up to 48 hours followed by a modest recovery.^139^ Treating healthy volunteers with an approved dose of LPS produced a dynamic plasma Lp‐PLA2 response according to unrelated studies, where the expression of Lp‐PLA2 mRNA displayed an initial decline, followed by an increase and finally recovered to the initial levels.^140^ Moreover, Claus et al^141^ found that patients with severe sepsis and other critical illnesses had reduced plasma Lp‐PLA2 activity on the day of intensive care unit admission compared with healthy controls, while the activity significantly increased over time irrespective of whether the clinical situation improved or the patients died. On the basis of this observation, Dr Stafforini speculated that the patients’ ability to maintain Lp‐PLA2 expression within a limited dynamic range, namely, the “expression window,” was a crucial determining factor in response to endotoxemia, and out of the “window” in either direction might be detrimental.^60^

In addition, recent research revealed quite a few potential regulators of Lp‐PLA2 expression. Nitro‐oleic acid (OA‐NO2), a nitrated fatty acid, might downregulate Lp‐PLA2 expression in THP‐1‐derived macrophages through multiple signaling pathways.^142^ Apolipoprotein CIII (Apo CIII) is one of the major components of TG‐rich lipoproteins and is chiefly synthesized in the liver. In vitro, Apo CIII could induce Lp‐PLA2 expression via MAPK and NFĸB pathways. Moreover, blood from Apo CIII transgenic pigs displayed a fourfold increase in plasma Lp‐PLA2 activity and a 10‐fold increase in Lp‐PLA2 mRNA in macrophages.^143^ Narahara et al^127^ did not detect any effect of insulin on Lp‐PLA2 secretion from TPA‐stimulated HL‐60 cells, whereas Schliefsteiner et al^144^ demonstrated that insulin could augment Lp‐PLA2 activity in human placental macrophages (Hofbauer cells and HBCs) and that leptin also led to a moderate yet significant upregulation. Simvastatin tends to inhibit Lp‐PLA2 expression and secretion activity in LPS‐stimulated human monocyte‐derived macrophages via inhibition of the mevalonate‐geranylgeranyl pyrophosphate (GGPP)‐RhoA‐p38 MAPK pathway.^145^ Serum amyloid A (SAA) is secreted primarily by hepatocytes and its level rapidly increases in humans after acute inflammatory stimuli. Li et al^146^ detected significantly upregulated expression of Lp‐PLA2 upon SAA treatment both in vitro and in vivo, and seemingly, the activation of peroxisome proliferator‐activated receptor‐γ (PPAR‐γ) pathways was involved in the process. Apart from PPAR‐γ, activation of PPAR‐α would generate a robust increase in Lp‐PLA2 mRNA expression in murine hepatocytes, which subsequently contributed to the production and secretion of 1‐palmitoyl lysoPC.^146^ Additionally, Du et al^147^ indicated that VLDL receptor deletion in mice probably caused diminished expression of Lp‐PLA2 from macrophages, which was mediated by Reln (Reelin, a VLDLR ligand) and Dab2 (disabled‐2, a cytoplasmic adapter protein).^147^

Altogether, the differences in the severity of the challenge, phase of the disease, susceptibility to stimulation, and state of cellular activation or differentiation might account for the diverse results. Hence, other influencing factors should also be considered when making specific comparisons. Although a wide spectrum of factors affects the Lp‐PLA2 expression, inflammation seems to play an important role in the regulatory mechanism. Likely, proinflammatory stimuli induce Lp‐PLA2 expression while anti‐inflammatory agents inhibit Lp‐PLA2 expression, and the dynamic alteration of Lp‐PLA2 levels possibly reflects different inﬂammatory states in vivo. However, the presence of many exceptions presents a challenge in affirming this conclusion. In addition, the complex inflammatory regulation system of living organisms further complicates the reproduction of the results from in vitro to in vivo. Furthermore, as Lp‐PLA2 circulates by binding to lipoproteins and participates in the regulation of lipid metabolism, its expression is also affected by lipid homeostasis.

## 4 BIOLOGICAL FUNCTIONS AND DISEASE IMPLICATIONS

Lp‐PLA2 was shown to have the ability to degrade the PAF and its analogs and the oxLDL species containing short, oxidized, and/or truncated *sn*‐2 chains, without any threat to the integrity of phospholipid components within cellular membranes and lipoproteins.^48^ In this context, Lp‐PLA2 might exert anti‐inflammatory effects, as PAF and its analogs and truncated oxLDL potentially induce inflammatory responses.^148^, ^149^, ^150^ However, increasing evidence suggests that Lp‐PLA2 seems to be a promoter in the development and progression of inflammation, primarily due to its proinflammatory products, lysoPC and oxNEFA, as previously described.^11^, ^13^ In addition, the binding of Lp‐PLA2 to various lipoproteins likely generates distinct physiological responses. For example, LDL‐associated and Lp(a)‐associated Lp‐PLA2 display similar proinflammatory and atherogenic activities, while HDL‐associated Lp‐PLA2 may exert anti‐inflammatory and antiatherogenic effects.^151^ However, the biological consequences of Lp‐PLA2 bound to different lipoproteins has not been adequately studied. The vast majority of known studies focused on alterations of plasma Lp‐PLA2 activity, which mostly reflected the fluctuation of LDL‐associated Lp‐PLA2. Nonetheless, to identify the definite role of Lp‐PLA2 in pathogenesis, several types of diverse diseases have been explored in animal models or clinical trials.

### 4.1 Atherosclerosis

Atherosclerosis, the underlying cause of the majority of clinical cardiovascular events, is a systemic disease process involving a combined effect of inflammation and immunological factors.^2^ The deposits and oxidation of LDL particles are built up within the walls of arteries during the atherosclerotic process. Incidentally, by inactivating potentially dangerous oxLDL, Lp‐PLA2 apparently plays an antiatherogenic role. Similarly, in vivo studies in animal models demonstrated that augmented plasma Lp‐PLA2 levels might alleviate atherosclerotic progression. Furthermore, subjects with the loss‐of‐function mutation (V279F) possibly suffer from greater incidence and severity of cardiovascular conditions compared with noncarriers.^11^ However, other lines of evidence suggest that Lp‐PLA2 contributes to the development and progression of atherosclerosis. The proatherogenic role of Lp‐PLA2 was postulated to be due to the proinflammatory products of Lp‐PLA2 activity on oxidized phospholipids, lysoPC and oxNEFA, which might promote atherosclerotic plaque development and eventually lead to the formation of a necrotic core. Increased levels of Lp‐PLA2 and lyso‐PC are found in thin‐cap fibroatheromas and ruptured plaques but are almost absent in stable lesions.^15^ Additionally, a deleterious feed‐forward mechanism may be involved in the generation of these proinflammatory mediators by Lp‐PLA2, whereby recruitment of macrophages, T‐cell lymphocytes, and mastocytes in activated plaques may result in further Lp‐PLA2 production and activity.^13^ Furthermore, many large‐scale epidemiological studies have found that elevated plasma Lp‐PLA2 levels are associated with an increased risk of coronary disease, stroke, and/or mortality, further substantiating the proatherogenic role of Lp‐PLA2.^152^ In addition, downregulating the expression of the *PLA2G7* gene by RNA interference could induce ameliorative inflammation and atherosclerosis in apolipoprotein E‐deficient mice.^153^ Taken together, these findings indicate that Lp‐PLA2 might be crucial during the development of atherosclerosis and in determining plaque instability via inflammatory pathways.

Considering its potential proatherogenic role, inhibition of Lp‐PLA2 might retard atherogenic progression. Consequently, a number of Lp‐PLA2 inhibitors were discovered, among which, darapladib (developed by GSK) is the most advanced and most widely studied Lp‐PLA2 inhibitor.^154^ As a potent oral inhibitor of Lp‐PLA2, darapladib was used as a candidate drug to elucidate the influence of Lp‐PLA2 inhibition on a range of cardiovascular and CVDs, including stroke, myocardial ischemia, subclinical atherosclerosis, CHD, and heart failure.^155^, ^156^ Recently, Campos et al^157^ extensively summarized the major preclinical and clinical studies on darapladib for atherosclerosis. In particular, two large phase III trials, the stabilization of atherosclerotic plaque by initiation of darapladib therapy (STABILITY) trial and the stabilization of plaques using darapladib‐thrombolysis in myocardial infarction 52 (SOLID‐TIMI 52) trial, failed to meet their primary endpoints, viz, decreasing the risk of cardiovascular death, heart attack or stroke and reducing CHD death, heart attack or urgent coronary revascularization respectively. More than 15 000 patients with stable CHD participated in the STABILITY trial, and after a median follow‐up of 3.7 years, no significant impact was detected on the incidence of major adverse cardiovascular events, which occurred in 9.7% of patients in the darapladib group and 10.4% of patients in the placebo group (HR = 0.94, 95% CI: 0.85‐1.03, *P* = .20).^20^ In the SOLID‐TIMI 52 trial, 13 026 subjects were selected at random to receive either once‐daily darapladib or placebo within 30 days of hospitalization with an acute coronary syndrome. With a median duration of 2.5 years, major coronary events occurred in 16.3% of patients in the darapladib group and 15.6% in the placebo group (HR = 1.00, 95% CI: 0.91‐1.09, *P* = .93).^19^

Several limitations were proposed to explain the failure of the previous clinical trials. For example, nearly 95% of the patients received statins as the basic treatment, which was associated with two indefinite concerns. One concern was that the efficacy of Lp‐PLA2 inhibitors in improving the outcomes in well‐managed statin‐treated patients was limited, as statins could not only inhibit plasma Lp‐PLA2 levels but also could reduce the occurrence of CHD events.^158^ A different concern was the rationality of the inclusion criteria for the population under study. Even after intensive statin therapy, many atherosclerosis patients suffered high rates of recurrent cardiovascular events. These patients could be classified into two categories: one represents a group with residual cholesterol risk (high sensitivity C‐reactive protein (hsCRP) <2 mg/L and low‐density lipoprotein cholesterol (LDL‐C) ≥70 mg/dL), and the other presented a residual inflammatory risk (hsCRP ≥2 mg/L and LDL‐C <70 mg/dL).^159^ Recently, canakinumab, an anticytokine agent targeting IL signaling pathways, was demonstrated to significantly reduce cardiovascular event rates following statin therapy, signifying a proof of principle that reducing inflammation in the absence of lipid lowering could diminish vascular event rates. In particular, the studied population in that clinical trial comprised stable postmyocardial infarction patients with hsCRP levels ≥2mg/L.^160^ However, the inclusion criteria of the SOLID‐TIMI 52 and STABILITY trials did not involve the hsCRP levels. Consequently, we supposed that patients with the residual inflammatory risk might benefit from Lp‐PLA2 inhibition. Furthermore, the possibility that Lp‐PLA2 might not be a suitable target to address the residual inflammatory risk could not be excluded, based on the observation that broad‐spectrum anti‐inflammatory therapy by methotrexate failed to reduce cardiovascular events while specific cytokine inhibition by canakinumab could.^161^ Additionally, the study failed to identify the beneficial effects of treatment on patients with higher baseline levels of Lp‐PLA2 activity, as patients were screened based on their Lp‐PLA2 activity levels.^19^, ^20^ Also, the effects of prolonged Lp‐PLA2 inhibition could not be determined since only 3 to 4 years of median follow‐up were recorded.^77^ Nevertheless, the failure of these clinical trials definitely dims the future of Lp‐PLA2 inhibitors as therapeutics for atherosclerosis but does not limit their potential to address other diseases.

Currently, Lp‐PLA2 is considered a reliable biomarker of cardiovascular risk, and many reports have established Lp‐PLA2 activity as an independent predictor of CHD outcomes in the general population.^162^, ^163^, ^164^, ^165^, ^166^, ^167^, ^168^, ^169^, ^170^, ^171^, ^172^ Since the vascular specificity of Lp‐PLA2 is different from conventional risk factors, in 2014, the Food and Drug Administration approved the Lp‐PLA2 test for patients without existing coronary disease measure a person's risk of heart disease, cardiac arrest, and the potential for other heart problems.^166^ Furthermore, the European Society of Cardiology recommended Lp‐PLA2 as part of a refined risk assessment in patients at high risk of a recurrent acute atherothrombotic event, and the American Heart Association/American Stroke Association recommended that measurement of the Lp‐PLA2 in patients without CVD might be helpful in identifying patients at an increased risk of stroke.^169^ Very recently, a pharmacogenetic (PGx) study was conducted to assess the influence of genetic variants on efficacy and tolerability in the aforementioned two trials. An analysis of darapladib efficacy endpoints was performed on data from more than 23 000 subjects referring to approximately 9.3 million common and low frequency variants. However, the efficacy analysis identified only one common locus (rs181937009), which conferred risk in the placebo arm but was protective in the darapladib treatment arm. Moreover, none of the variants that were significantly associated with baseline Lp‐PLA2 activity was associated with efficacy endpoints.^38^ This finding was validated in another genetic study with 261 950 total participants, which identified that both Lp‐PLA2‐lowering alleles and darapladib treatment were not related to CHD risk.^77^ Altogether, this evidence indicates that Lp‐PLA2 is a reliable biomarker rather than a causal risk factor for CVDs, but this view remains to be confirmed.^173^

### 4.2 Alzheimer's disease

AD, pathologically characterized by the deposition of amyloid β (Aβ) peptide and neurofibrillary tangles formed by tau proteins in the brain, is the most common cause of dementia in elderly individuals.^174^ Breakdown of the BBB, a highly specialized brain endothelial structure of the fully differentiated neurovascular system, seems to be involved in the etiopathology of AD. In conjunction with astrocytes and microglia, the BBB elaborately regulates the exchange of substances between the blood and brain to uphold the microenvironment homeostasis of the CNS.^175^ In particular, the tight junctions between the brain vascular endothelial cells (BVECs), comprising occludin proteins, claudin proteins, and junctional adhesion molecules, are vital for the structural and functional integrity of BBB.^176^ Therefore, BBB breakdown, due to disruption of the tight junctions, inflammatory responses, or other risk factors, may enhance its permeability and induce the leakage of harmful substances from blood vessels into the brain, contributing to the process of AD.^175^, ^177^ Very recently, Nation et al^178^ demonstrated that individuals with early cognitive dysfunction develop brain capillary damage and BBB breakdown in the hippocampus and established BBB breakdown as an early biomarker of human cognitive dysfunction in individuals with and without Aβ or phosphorylated tau (pTau) positivity. Therefore, the authors concluded that neurovascular dysfunction might be a previously underappreciated promotor of AD, independent of the classic pathophysiological hallmarks.^178^ In this context, Lp‐PLA2, a specific marker of vascular inflammation, was investigated to determine its role in AD.

The epidemiological investigations on the relationship between Lp‐PLA2 activity and AD yielded inconsistent results. Two studies demonstrated no significant association between plasma Lp‐PLA2 mass and AD.^179^, ^180^ In addition, Davidson et al^181^ observed that plasma Lp‐PLA2 activity was not associated with a diagnosis of AD, since no strong correlations were found between Lp‐PLA2 activity and cerebrospinal fluid (CSF) markers of AD. Furthermore, for the *PLA2G7* null mutation (V279F), Lp‐PLA2 activity was not associated with a diminished risk of AD in a Japanese population.^86^ However, several limitations might be responsible for the lack of association, such as the small size of AD cases, age at which the markers were tested, inadequate or overlong duration of follow‐up (this effect has been discussed in two studies),^182^, ^183^ lower levels of inflammation, and less attention to Lp‐PLA2 mass.^183^, ^184^ Nevertheless, in a case‐control study, the higher plasma levels of Lp‐PLA2 were found to be independently associated with AD and interact with cardiovascular disease equivalent to increase the risk of AD.^184^ Similarly, Fitzpatrick et al^185^ demonstrated Lp‐PLA2 to be an independent risk factor for dementia and AD through a comparison between the highest and lowest quartiles of Lp‐PLA2 mass (HR = 2.21, 95% CI: 1.12‐4.37; HR = 1.98, 95% CI: 1.22‐3.21, respectively). Considering the large enough sample, appropriate follow‐up duration, and refined classification, we believed that this investigation was effective to in detecting the association between Lp‐PLA2 and the risk of AD. Overall, though inclusive, Lp‐PLA2 is likely to be a risk factor for AD.

Initially, darapladib, a selective and orally effective Lp‐PLA2 inhibitor, was investigated for its ability to inhibit the development of advanced atherosclerotic lesions in a diabetic‐hypercholesterolemic (DMHC) porcine model. The researchers unexpectedly discovered that darapladib treatment resulted in improved alertness and activity in DMHC animals compared with untreated DMHC animals. In consideration of the increased BBB permeability of DMHC animals, they hypothesized that the observed behavioral improvements in darapladib‐treated animals were probably due to the beneﬁcial effects on the functional integrity of the BBB. To explore this speculation, they monitored the variation in BBB permeability in DMHC pigs after darapladib treatment for 6 months. Compared with the untreated group, the darapladib‐treated group showed significantly alleviation of the deleterious effects induced by prolonged diabetes mellitus (DM) and hypercholesterolemia (HC) in the brain of DMHC pigs. In particular, darapladib improved the compromised BBB permeability and reduced the inﬂux of plasma components into the brain tissue, which occurred principally in the arterioles within the microvasculature.^186^ A subsequently published study revealed that claudin‐5 and occludin, tight junction proteins in BVECs, might be involved in the altered BBB permeability.^36^ Using immunoglobulin G (IgG) as a visual indicator of local BBB breakdown and vascular leak, they established that the leaky microvascular IgG bound preferentially to pyramidal neurons in the cerebral cortex. Moreover, the authors also detected enhanced deposition of the Aβ42 peptide predominantly on the pyramidal neurons within the brain parenchyma of DMHC pigs, which are the same neurons in human AD brains that accumulate excessive amounts of Aβ42‐positive material. These two alterations within neuronal cells potentially induced functional disruption and pathological changes, which could be recovered to nearly normal levels using darapladib.^186^

A phase II trial, which was carried out to reproduce these findings in a relevant AD population, also demonstrated that Lp‐PLA2 inhibition could offer beneﬁcial effects on AD progression. The participants with AD and CVD were randomized to treatment with rilapladib, another potent Lp‐PLA2 inhibitor developed by GSK, or placebo once daily for 24 weeks on top of their stable background therapy. Compared with the placebo, rilapladib treatment resulted in low levels of plasma CSF neurodegenerative markers, including albumin quotient (AlbQ), total tau (T‐tau), 181‐pTau, and neurofilament light chain (NF‐L), although the difference was not significant. However, neither CSF nor plasma Aβ were considerably altered by the administration of rilapladib, which was not consistent with the observations in the nonclinical model. Nonetheless, statistically significant improvements were detected in the executive function/working memory (EF/WM) composite score (effect size (ES) = 0.45, 95% CI: 0.055‐0.84) and overall composite score (ES = 0.43, 95% CI: 0.032‐0.82) among the cognitive data. Further covariate interaction testing at week 24 for two primary endpoints of interest, CSF Aβ_1‐42_ and CogState executive EF/WM composite score, failed to identify any significant association. Collectively, even though the epidemiological results could not determine the role of Lp‐PLA2 in dementia, the nonclinical and clinical studies attributed the alleviation of AD progression to Lp‐PLA2 inhibition. Remarkably, the beneficial effect of Lp‐PLA2 inhibitors might result from preventing BBB breakdown in a cerebral amyloidosis‐independent manner.^21^ Seemingly, this result provided evidence that targeting the BBB or neurovascular unit might alleviate the progress of AD and demonstrated the independence of the BBB breakdown pathway from the Aβ/tau pathway as described by Nation and coworkers.^178^

### 4.3 Diabetic retinopathy

Diabetic retinopathy (DR) is classified as a microvascular complication of DM, which is the major cause of partial and complete vision loss in diabetic patients. The features of DR include the loss of ECs and pericytes of retinal capillaries, breakdown and leakage of the blood‐retinal barrier (BRB), formation of acellular capillaries, and neovascularization.^187^ On the basis of the proliferative status of the retinal vasculature, DR has been divided into two stages: a nonproliferative stage (NPDR), characterized by microaneurysms, retinal hemorrhages, vascular tortuosity, and hard exudates; and a proliferative stage (PDR), characterized by the creation of new but leaky blood vessels.^188^ In addition, DME is a unique condition of DR, which is characterized by fluid accumulation in the neural retina DME can occur in both the NPDR and PDR phases and is considered the clinical feature that is most strongly associated with vision loss.^189^ Even though the pathology and molecular mechanism of DR are complicated, it is probably not only a vascular disease but also a neurodegenerative disease. Moreover, increasing evidence suggests that inflammation is a critical contributor to the development of DR via both vascular and neurosensory pathways.^188^, ^190^

Considering the potential proinflammatory effect of Lp‐PLA2, a number of studies were carried out to determine the role of this enzyme in DR. A recent epidemiologic study showed that the activity of plasma Lp‐PLA2 is higher in diabetic patients with PDR than in healthy individuals and diabetic patients with NPDR. In addition, the increase in Lp‐PLA2 was correlated with the severity of DR.^191^ Very recently, a longitudinal disease progression study was reported, aiming to explore the association among Lp‐PLA2 activity levels, incident DR, and change in retinopathy grade. In each analysis, the participants were divided into four equal‐sized groups based on their Lp‐PLA2 activity levels, and the lowest quartile (Q1) was regarded as the reference. Consequently, the authors established that the type 2 diabetic patients in the highest Lp‐PLA2 quartile (Q4) suffered an increased risk of mortality compared with the lowest quartile (HR = 1.45, 95% CI: 1.24‐1.68, *P* < .001). In survival analyses, the hazard of developing incident DR was amplified along with the increased plasma Lp‐PLA2 activity level compared with the lowest quartile, signifying a progressive trend of increased risk across quartiles in the 3‐year follow‐up period. In addition, higher Lp‐PLA2 activity levels were associated with a significantly increased risk as well as transitions to all grades. Through comparison of the highest quartile with the lowest quartile after a follow‐up of 3 years, the hazards of developing observable (or more severe) and referable (or more severe) retinopathy were 2.82 (95% CI: 1.71‐4.65, *P* < .001) and 1.87 (95% CI: 1.26‐2.77, *P* < .01), respectively. Furthermore, these associations were independent of the calculated LDL‐cholesterol and other traditional risk factors but were sensitive to the temporal proximity of death and retinopathy events to the time of serum collection and Lp‐PLA2 measurement. On the basis of these observations, the authors established that Lp‐PLA2 could be used to predict the incidence and progression of DR in a time‐sensitive manner.^16^

In a murine model of experimental autoimmune uveoretinitis (EAU), Lp‐PLA2 ablation through gene knockout or a pharmacological inhibitor failed to modify the onset or progression of EAU.^192^ On the other hand, deletion of Lp‐PLA2 might have prevented pericyte loss and other signs of DR microvasculopathy in a recent study of diabetic *PLA2G7* knockout mice.^193^ Furthermore, Lp‐PLA2 was identified as a valid therapeutic target for DR according to two studies conducted by Canning et al^37^ and Acharya et al^36^ on rat and pig, respectively, models of DR. Given the impact of darapladib on the BBB and the similarity between the BBB and BRB, both studies focused on the effect of darapladib‐mediated Lp‐PLA2 inhibition on alteration of the function and structure of the BRB. In the first study, Canning et al^37^ demonstrated that systemic Lp‐PLA2 inhibition by darapladib effectively reduced retinal vascular leakage in both a preventive and therapeutic manner in streptozotocin (STZ)‐induced diabetic Brown Norway rats. Moreover, the diminished retinal vasopermeability induced by inhibition of Lp‐PLA2 was independent of the vascular endothelial growth factor (VEGF) signaling axis, and intravitreal VEGF neutralization combined with darapladib treatment synergistically inhibited diabetes‐induced vasopermeability. Further mechanistic studies revealed that elevated plasma lysoPC and the production of Lp‐PLA2 could act on the luminal side of the endothelium to induce vascular leakage in diabetes, which required signaling by the VEGF receptor 2. Taken together, these findings indicate that Lp‐PLA2 might be a valuable VEGF‐independent therapeutic target for DR.^37^ The second study was performed on a porcine model with chronic DM and HC. Acharya et al^36^ described that darapladib treatment could maintain the layered architecture of retina, decrease the permeability of the BRB, limit the influx of plasma components including IgG into the retina, and downregulate the activation of Müller cells, all of which were alleviated and comparable to those of the healthy control group. Furthermore, the DMHC pigs exhibited a significantly higher incidence of IgG‐labeled neurons (ganglion cells) in the ganglion cell layer (GCL) of the retina compared with the controls. Together with the thinning GCL and decreased cell numbers, these observations further supported a causal link between BRB‐associated plasma influx, inflammation, and degenerative changes. Importantly, these findings also support that Lp‐PLA2 inhibitors, such as darapladib, might be valuable in eye diseases characterized by vascular abnormalities.^36^ Additionally, our group reported a novel Lp‐PLA2 inhibitor that could inhibit retinal thickening in STZ‐induced diabetic SD rats after oral dosing for 4 weeks, and further comprehensive studies are currently underway.^194^

To scrutinize the ability of an Lp‐PLA2 inhibitor to reduce edema and improve vision in subjects with center‐involved DME, a randomized, double‐masked phase IIa clinical trial was performed, in which darapladib 160 mg or placebo monotherapy was orally administered once daily for 3 months, and patients were followed up monthly for 4 months. Consequently, approximately 4 Early Treatment Diabetic Retinopathy Study (ETDRS) letters in best‐corrected visual acuity (BCVA) and 60 mm central subfield thickness improvements were observed in the darapladib treatment group, whereas these parameters were not significantly enhanced in the placebo group. In comparison, the laser and ranibizumab therapies in a study with similar baseline visual acuity over the same period provided approximately 2.5 and 7 letters (ETDRS) and 40 and 125 mm central subfield thickness improvements, respectively, Lp‐PLA2 inhibition demonstrated modest improvements in vision and macular edema. Altogether, these data provide preliminary evidence supporting Lp‐PLA2 inhibition as a novel therapeutic approach for DR.^22^

Though Lp‐PLA2 inhibition showed limited benefit for macroangiopathy in clinical trials, it might efficiently improve certain diseases with microvascular abnormalities, such as DR and AD. The results from the above two phase II clinical trials support the hypothesis that agents with vascular anti‐inflammatory properties can ameliorate the progression of diseases characterized by impaired BBB and BRB permeability. Undeniably, these findings need to be confirmed in large clinical trials of long duration, along with comprehensive studies on the pathogenic mechanism, pharmacogenetics, racial specificity, or disease subtype specificity. In general, these findings encourage researchers to develop new therapeutic approaches for chronic neurodegenerative disorders of the BBB.

### 4.4 Cancers

The role of Lp‐PLA2 was also investigated in various cancer types. Aberrant Lp‐PLA2 expression exists in a variety of cancers, and most tumor tissues show higher level compared with normal tissues. Moreover, the invasive and metastatic tumor samples exhibited greater *PLA2G7* expression compared with the primary tumor samples, which were derived from the breast, colon, kidney, liver, or lung.^18^ In terms of pathogenic mechanisms, both pro‐ and antitumorigenic effects have been described for Lp‐PLA2. Seemingly, the net effects of Lp‐PLA2 on tumors and tumor microenvironments result from the combined actions of its substrates, products, and secondary products. Previously, Stafforini^195^ reviewed the potential biological consequences of altered Lp‐PLA2 expression or function. By regulating the metabolism of biologically active phospholipids, Lp‐PLA2 might control all aspects of tumor progression, such as tumor cell proliferation, apoptosis, invasion, metastasis, and angiogenesis. Nevertheless, numerous studies have demonstrated the role of Lp‐PLA2 in tumorigenesis through nonspecific inhibition, overexpression, and exogenous supplementation under diverse experimental conditions.^195^ Thus, acquiring roughly consistent results across studies is difficult, especially in consideration of the complex tumor microenvironment. Nonetheless, these data provide initial evidence that Lp‐PLA2 takes part in the pathologic process of certain cancers.

Several studies have investigated the effects of Lp‐PLA2 on specific tumor types in vitro or in vivo. Vainio et al^196^ observed that *PLA2G7* overexpression was present in a majority of clinical prostate tumors and correlated positively (*r* = 0.66, *P* < .001) with the expression of *ERG*, an E twenty‐six gene (*ETS*)‐related gene that may be involved in the initiation and progression of prostate cancer. Further functional studies revealed that silencing *PLA2G7* expression produced antiproliferative, proapoptotic, and antimigratory effects on prostate cancer cells and that statin treatment combined with *PLA2G7* silencing synergistically exerted an antiproliferative effect.^197^ Recently, Alinezhad et al^198^ also identified *PLA2G7* as a valid biomarker for prostate cancer and subsequently evaluated its functions in disease‐relevant processes. Similarly, diminished tumor cell invasion and proliferation were detected after silencing *PLA2G7*. In addition, the *PLA2G7* gene was found to be significantly associated with altered phospholipid metabolism in prostate cancer, signifying a key role in the pathogenesis.^198^ Overall, these observations indicate that Lp‐PLA2 might be a valuable biomarker or therapeutic target for prostate cancer, particularly, in *ERG*‐positive prostate cancers.

Lp‐PLA2 activity and mRNA levels in tumor tissues and plasma from colon cancer patients were higher than those in healthy controls. The increased *PLA2G7* expression might be synergistically controlled by loss‐of‐function p53 and Ras activation in murine and human colon cells, and silencing *PLA2G7* could decrease the size of the tumor by 42% in vivo.^199^ Furthermore, Xu et al^200^ demonstrated that *PLA2G7* knockout *Apc* ^Min/+^ mice had robust suppression of intestinal polyposis and colon tumor formation. These data suggest a causal role for Lp‐PLA2 in colon tumorigenesis, and Lp‐PLA2 inhibition might be a promising approach to treat intestinal malignancies.^200^ Additionally, a number of studies have elucidated the role of Lp‐PLA2 in breast cancer. Earlier, Kispert et al demonstrated that Lp‐PLA2 inhibition by cigarette smoke extract induced cell motility in MDA‐MB‐231 cells and increased its adherence to the lung endothelium.^201^, ^202^ However, a recent study revealed possible beneficial effects of Lp‐PLA2 inhibition in advanced breast cancer, such as downregulation of epithelial‐mesenchymal transition (EMT) as well as cell migration and invasion. Furthermore, *PLA2G7* was found to be overexpressed in hormone receptor‐negative breast cancers and high *PLA2G7* mRNA expression was associated with poor prognosis in primary breast cancer samples.^18^ In addition, high Lp‐PLA2 protein and gene expression were significantly correlated with poor prognosis in lymph node metastases. Together with high *PLA2G7* expression in four other metastatic or aggressive tumors, namely, colon, kidney, liver, and lung cancer, Lp‐PLA2 exerted a wider range of significance in cancer progression.^18^ Hence, clarifying the role of Lp‐PLA2 in cancer progression and even the effectiveness of Lp‐PLA2 as a therapeutic target to manage cancers is meritorious.

A few reports in the literature described the relationships between Lp‐PLA2 and other types of tumors. To establish the associations between lipid mediators in the tumor microenvironment and the clinical outcome of high‐grade serous ovarian carcinoma, Reinartz et al^203^ detected levels of cytokines and lipids in ascites before first‐line therapy. Kaplan‐Meier analysis revealed that the protein level of Lp‐PLA2 in ascitic fluid was inversely related to relapse‐free survival. Protumorigenic tumor‐associated macrophages (TAMs) seemed to be involved in this process since they express *PLA2G7* at higher levels than tumor cells.^203^ Interestingly, lysoPC16:0, the major product of Lp‐PLA2, was increased in the plasma of early‐stage epithelial ovarian cancer patients while other lysoPCs were decreased, suggesting that Lp‐PLA2 might play an important role in the development of ovarian cancer.^204^ Similarly, TAM‐derived Lp‐PLA2 was found to enhance the migration of nasopharyngeal carcinoma cells, possibly stimulating tumor invasiveness or metastasis.^205^ Altogether, these results indicate that TAMs might exert a tumor‐promoting function in various cancers and targeting TAMs, such as inhibiting the induction of *PLA2G7* expression, is beneficial for addressing the tumor process. In addition, *PLA2G7* gene expression in peripheral blood might be able to predict the response to immunotherapy among melanoma patients, in combination with the expression of three additional genes including cathepsin D (*CTSD*), thioredoxin reductase 1 (*TXNRD1*), and interleukin 1 receptor‐associated kinase 3 (*IRAK3*). The ability of *PLA2G7* to improve the predictive power of the gene signatures probably results from the regulation of the tumor microenvironment, and an elevated level of *PLA2G7* was a predictor of increased survival following anti‐cytotoxic T lymphocyte‐associated antigen‐4 directed therapy.^206^ Owing to the autoimmune toxicity induced by immunotherapy, the discovery of the biomarkers, especially blood biomarkers such as *PLA2G7*, is valuable and could aid the identification of the subset of patients who can benefit from immunotherapy and simultaneously exhibit low risk for autoimmune toxicity.^207^, ^208^ Furthermore, *PLA2G7* was also identified as a diagnostic marker via a microarray technique for profiling the transcriptome of esophageal cancer cells. It was significantly overexpressed in earlier tumor stages of both histological subtypes, namely, esophageal squamous cell carcinoma and esophageal adenocarcinoma.^209^ Due to its potential in promoting cancer cell migration and invasion, the authors speculated that Lp‐PLA2 might be a promising anticancer target.

Although significant efforts are needed for establishing the protumorigenic role of Lp‐PLA2 in some types of cancer, the results of the existing studies proposed a potential approach to managing cancers through Lp‐PLA2 inhibition. Certainly, the approach is challenging since Lp‐PLA2 might have a beneficial role in certain cancers such as melanoma, multiple myeloma, and glioblastoma. In this context, precision medicine might be helpful for identifying specific cancer subtypes that could be alleviated by inhibiting Lp‐PLA2.^210^ Furthermore, this approach might maximize anticancer effects and minimize undesirable side effects by locally downregulating the Lp‐PLA2 level in tissues and organs that overexpress it during tumorigenesis. Importantly, different types of cancers harbor different levels of *PLA2G7* mutations; thus, the effects of *PLA2G7* mutations should be taken into consideration when determining the role of Lp‐PLA2 in the pathogenesis of certain cancers. In addition to being a therapeutic target, Lp‐PLA2 has the potential to become a valuable biomarker for both predictive and prognostic applications in some cancers, such as breast cancer, glioblastoma, melanoma, and esophageal cancer. Combined with the straightforward acquisition and measurement, Lp‐PLA2 shows promising applied value in clinical practice. Altogether, an urgent need exists for comprehensive studies to furtherly determine the role of Lp‐PLA2 in cancer progression and the putative potential of Lp‐PLA2‐targeted therapy in cancer management.

### 4.5 Other diseases

In addition to the impact of Lp‐PLA2 on traditional CVDs, its role is also determined for various diseases in which CVD is a complication. Similar to DR, significantly altered Lp‐PLA2 levels were observed in diabetic macroangiopathy. Lp‐PLA2 was significantly associated with fasting plasma glucose and cardiovascular outcome in diabetic patients.^211^ Together with five other variables, Lp‐PLA2 showed significantly improved power for predicting 10‐year cardiovascular survival in patients with DM compared with the widely used algorithm (UK Prospective Diabetes Study [UKPDS]).^212^ Furthermore, positive correlations between Lp‐PLA2 and several atherogenic factors in patients with newly diagnosed diabetes as well as the cardio‐ankle vascular index, a recently developed marker of arterial stiffness, in long‐term T2DM patients were demonstrated recently.^213^ Lin et al^214^ proposed that Lp‐PLA2 might affect the incidence of atherosclerosis in diabetic patients and the decline in Lp‐PLA2 levels through intensive insulin therapy might contribute to protection from diabetic atherosclerotic complications.^214^ Additional studies are essential to clarify the role of Lp‐PLA2 in modifying the atherosclerotic process of T2DM patients.

Macrophages and phospholipases are assumed to play a key role in immune activation and inﬂammation in the context of human immunodeficiency virus (HIV) infection.^215^, ^216^ Notably, HIV‐infected patients exhibit increased arterial inflammation that persists even after effective antiretroviral therapy (ART), suggesting the need for adjunctive, anti‐inflammatory strategies to prevent and reduce CVD in virally suppressed HIV sufferers.^217^, ^218^ The Lp‐PLA2 level is unusually high in HIV‐infected patients and is further elevated after ART or protease inhibitor‐based treatment. Furthermore, Lp‐PLA2 is associated with several risk factors for HIV‐associated CVD, such as carotid intima‐media thickness (cIMT) and CAC.^219^ Among HIV‐infected individuals, statin therapy that can reduce the Lp‐PLA2 level could lead to a decline in specific markers of immune activation and arterial inflammation, noncalcified plaque volume, and high‐risk coronary plaque features.^220^ Hence, Lp‐PLA2 might be a suitable predictor of subclinical atherosclerosis or even a possible pharmacologic target to prevent CVD in HIV‐infected individuals, but comprehensive studies are obligatory to determine the effectiveness of Lp‐PLA2 in the clinical setting for HIV‐infected patients.

Lp(a) appears to be a causal risk factor for CVD and calcific aortic valve disease (CAVD),^221^ and Lp‐PLA2 and oxidized phospholipids might be important determinants of the functionality and pathophysiological role of Lp(a).^151^ Therefore, targeting the Lp(a)‐Lp‐PLA2–OxPL axis might be a viable approach for treating CAVD.^222^ Chronic periodontitis (CP), which can lead to elevated Lp‐PLA2 levels, is most likely associated with atheroma formation and cardiovascular events among CP subjects.^223^ In addition, periodontal treatments that can significantly decrease the Lp‐PLA2 level may result in a downregulation of the systemic inflammatory response, most likely decreasing the risk of CVD development.^224^, ^225^ Therefore, additional studies that determine the effects of Lp‐PLA2 inhibition alone or in combination with periodontal treatments on the risk of CVD in CP patients are valuable for the control of CVD. Additionally, both positive and negative associations between plasma Lp‐PLA2 levels and nonalcoholic fatty liver disease (NAFLD) were demonstrated in epidemiological studies, and Lp‐PLA2 seemingly protected subjects against the initiation and progression of NAFLD.^226^, ^227^ However, considering the observation that lysoPC administration could induce hepatitis in vivo, contrary to the protective role of Lp‐PLA2,^228^ further investigations using a longitudinal design is necessary to identify the cause.

In recent years, the role of Lp‐PLA2 as a prognostic or diagnostic biomarker has also been evaluated for many diseases. For “biomarker orphan” diseases, such as Rett syndrome and autism spectrum disorders (ASDs), Lp‐PLA2, which showed a significant inverse association only with individuals with ASDs compared with healthy controls, was identified as a novel serum biomarker for their differential diagnosis.^229^ The increased Lp‐PLA2 level in both obstructive sleep apnea (OSA) and obesity might adversely affect endothelial integrity, which decreased after OSA treatment. Thus, plasma Lp‐PLA2 activity levels may provide a reliable biomarker for children at risk for underlying atherosclerosis and vascular dysfunction atherosclerosis in the presence of obesity or OSA.^230^ Similarly, plasma Lp‐PLA2 activity is predominantly upregulated in patients with antiphospholipid antibodies, suggesting a prognostic biomarker role for Lp‐PLA2 in managing the antiphospholipid syndrome.^231^ In a cohort of rheumatoid arthritis (RA) patients, the baseline Lp‐PLA2 mass was significantly associated with both disease severity and subclinical atherosclerosis including IMT and flow‐mediated dilation at both baseline and follow‐up. Consequently, Lp‐PLA2 might be a biomarker of vascular damage among patients with RA.^232^ In addition, a prospective longitudinal cohort study identified the plasma Lp‐PLA2 activity and mass, which were independently and significantly associated with incident abdominal aortic aneurysm (AAA), as markers of AAA risk.^17^

Certain relationships between Lp‐PLA2 and these diseases were already detected; however, further research is needed for a deeper understanding of the associations. Comprehensive pathophysiological, epidemiological, and genetic studies would be unquestionably helpful for determining the diagnostic and therapeutic utility of Lp‐PLA2 in various diseases.

## 5 IDENTIFICATION OF SMALL‐MOLECULE INHIBITORS OF LP‐PLA2

Synthetic Lp‐PLA2 inhibitors have been developed for more than 20 years to explore their potential application in treating various diseases. Since being granted by Human Genome Sciences in 1993, GSK has consistently led the discovery of small‐molecule inhibitors of Lp‐PLA2, and three compounds have successively progressed to clinical research. Likewise, our group has focused on the identification of novel Lp‐PLA2 inhibitors and their role in various disease models as well. Remarkably, utilizing the FBLD approach, both Astex cooperating with GSK and our group have recently discovered novel Lp‐PLA2 inhibitors with new skeleton and promising prospects for research. Interest in the development of Lp‐PLA2 inhibitors has since become a topic of intense interest. In addition, many other groups also showed interest in the development of Lp‐PLA2 inhibitors and reported numerous compounds with diverse structures in past decades.

PLA2‐VIIB, also referred to as PAF‐AH II, is a 40 kDa intracellular phospholipase possessing an N‐terminal myristoylation site. Because of its 41% sequence homology and conserved catalytic site with Lp‐PLA2, PLA2‐VIIB shows similar substrate specificity.^11^ Although the toxicological consequences of PLA2‐VIIB inhibition remain unclear, a preferable Lp‐PLA2 inhibitor is predicted to possess over 100‐fold selectivity against PLA2‐VIIB.^35^

### 5.1 PAF analogs

**Figure 8:**
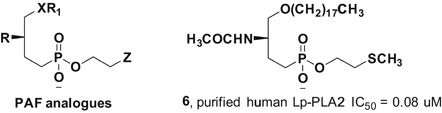
The structure of PAF analogs. IC_50_ values were determined by measuring the rate of turnover of the artificial substrate 1‐decanoyl‐2‐(4‐nitrophenylglutaryl) phosphatidylcholine. Assays were performed in 96‐well microtitre plates using purified human plasma Lp‐PLA2. Lp‐PLA2, lipoprotein‐associated phospholipase A2; PAF, platelet‐activating factor

As PAF can be effectively metabolized by Lp‐PLA2, an early study focused on the identification of PAF analogs that bind to Lp‐PLA2 and compete with its endogenous substrates. In the general molecular formula, R represents an acetamide or trifluoroacetamide, Z contains a high polar moiety including a quaternary ammonium group, and a XR1 is a long‐chain fatty ether (Figure 8). Among the published compounds, **6** showed the best inhibitory activity with an IC_50_ value of 0.08 μM for the purified plasma Lp‐PLA2, and an IC_50_ value of 30 μM for the inhibition of elevated lysoPC during Cu^2+^‐induced LDL oxidation.^233^

### 5.2 Pyrimidone derivatives

**Figure 9:**
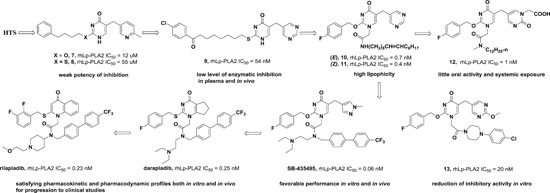
The discovery of darapladib and rilapladib. IC_50_ values were determined by measuring the rate of turnover of the artificial substrate DNPG. Assays were performed in 96‐well microtitre plates using recombinant human Lp‐PLA2. DNPG, 1‐decanoyl‐2‐(4‐nitrophenylglutaryl)phosphatidylcholine; HTS, high throughput screening; Lp‐PLA2, lipoprotein‐associated phospholipase A2

Initially, GSK obtained two weak Lp‐PLA2 inhibitors, **7** and **8** (IC_50_ = 12 and 55 μM, respectively), through high throughput screening (HTS) of their compound bank. On the basis of the lead **7**, they determined the impact of substituents at positions 2 and 5 of the pyrimidone on Lp‐PLA2 activity, consequently identifying a potent inhibitor **9** (IC_50_ = 0.054 μM). However, further studies showed that **9** weakly inhibits Lp‐PLA2 in plasma and in vivo. To improve these properties, a series of N‐1 substituted pyrimidin‐4‐one derivatives were synthesized, and compounds **10** and **11** demonstrated good levels of orally active inhibition (over 80% of the maximum inhibition) with a promising duration of action (≥5 hours) in Watanabe heritable hyperlipidemic (WHHL) rabbits at 10 mg/kg.^234^ Hydrophilic fragments were then introduced at position 5 of the pyrimidone based on compounds **10** and **11** to ameliorate water solubility at physiological pH. As a result, **12** exhibited not only a high solubility (>15 mg/mL) in normal saline but also a favorable profile following infusion dosing, including a promising concentration and enzymatic inhibition in the blood and enhanced CNS penetration.^235^ Subsequently, to further improve the druggability of these pyrimidine derivatives, the long lipophilic chain of the N‐1 substituent was replaced with other inhibitors including piperazine amide (compound **13**)^236^ and biarylmethyl amide moieties (SB‐435495 and darapladib).^237^, ^238^ Together with the introduction of novel substituents at position 5 of the pyrimidone ring, SB‐435495 and darapladib were selected for further clinical evaluation. Both SB‐435495 and darapladib showed excellent inhibitory activities (IC_50_ = 0.06 and 0.25 nM, respectively) in recombinant human Lp‐PLA2 (rhLp‐PLA2) assays, high oral performance at 10 mg/kg in WHHL rabbits (50% inhibition for ≥ 8 hours), and favorable pharmacokinetic (PK) and safety properties. The compounds were therefore studied in future clinical trials with a particular focus on darapladib, due to its enhanced in vitro and in vivo profiles vs SB‐435495. Furthermore, through bioisosteric replacement of the pyrimidone ring, a further inhibitor (rilapladib) with good PK and pharmacodynamic (PD) profiles entered into clinical studies (Figure 9).^239^ The preliminary human experimental evaluation demonstrated that the highest clinical dose of darapladib (80 mg nonenteric coated or 160 mg enteric coated) could maintain approximately 80% inhibition of plasma Lp‐PLA2 levels 24 hours after administration. At the same 24‐hour postdose time point, the top clinical dose of rilapladib (250 mg enteric coated) generated an even greater magnitude of inhibition of approximately 90%, with essentially complete inhibition determined at earlier time points. Furthermore, both darapladib and rilapladib were well tolerated with no major side effects observed. Both had no influence on platelet aggregation and displayed a good correlation of PD and PK effects.^240^ Subsequently, both compounds have been under assessment in further clinical trials to evaluate the possibility of Lp‐PLA2 as a therapeutic target for various diseases.

**Figure 10:**
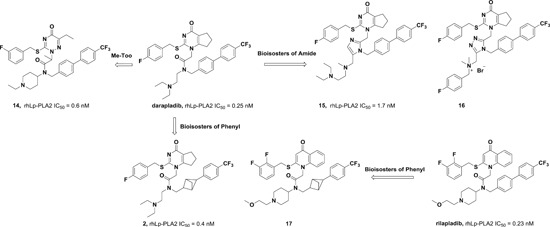
The structures of reported darapladib analogs. The IC_50_ of compound **14** values was determined by measuring the rate of turnover of the substrate 1‐*O*‐hexa decyl‐2‐deoxy‐2‐thio‐*S*‐acetyl‐*sn*‐glyceryl‐3‐phosphorylcholine (2‐thio‐PAF). The IC_50_ values of compounds **2**, darapladib and rilapladib were determined by measuring the rate of turnover of the artificial substrate DNPG. The IC_50_ value of compound **15** was determined by measuring the rate of turnover of the substrate [ ^3^H]PAF. Assays were performed in 96‐well microtitre plates using recombinant human Lp‐PLA2. HTS, high throughput screening. DNPG, 1‐decanoyl‐2‐(4‐nitrophenylglutaryl)phosphatidylcholine; Lp‐PLA2, lipoprotein‐associated phospholipase A2; PAF, platelet‐activating factor

Several darapladib analogs have now been reported (Figure 10). Bayer Company used the triazinone structure to mimic the pyrimidine in darapladib and obtained a series of compounds with good inhibitory in vitro potency. In particular, the IC_50_ of compound **14** against rhLp‐PLA2 was 0.4 nM.^241^ Our group also developed two types of darapladib analogs through bioisosteric replacement of the amide group of darapladib with an imidazole (**15**) or a triazole (**16**). Following further optimization, we identified a number of orally bioactive Lp‐PLA2 inhibitors in C57BL/6 mice.^242^, ^243^ Recent studies reported that a bicyclo[1.1.1]pentane moiety replacement of a phenyl ring in darapladib and rilapladib maintained inhibitory potency and displayed improved physicochemical properties compared to their respective parent compounds. This substitution resulted in improved solubility and an enhanced overall PK profile.^68^

**Figure 11:**
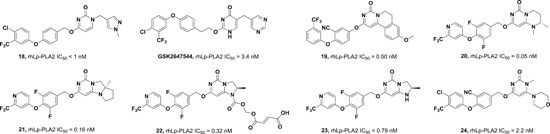
The representative structures of novel pyrimidine Lp‐PLA2 inhibitors. The IC_50_ values of compounds **18**, **19**, **20**, **21**, and GSK2647544 were determined by measuring the rate of turnover of the substrate PED6. The IC_50_ values of compounds **22**, **23**, and **24** were determined by measuring the rate of turnover of the substrate 2‐thio‐PAF. Assays were performed in 96‐well microtitre plates using recombinant human Lp‐PLA2. Lp‐PLA2, lipoprotein‐associated phospholipase A2; PAF, platelet‐activating factor; PED6, *N*‐((6‐(2,4‐dinitrophenyl)amino)hexanoyl)‐2‐(4,4‐difluoro‐5,7‐dimethyl‐4‐bora‐3a,4a‐diaza‐*s*‐indacene‐3‐pentanoyl)‐1‐hexadecanoyl‐*sn*‐glycero‐3‐phosphoethanolamine, triethylammonium salt

However, although darapladib can be used orally, it displayed poor plasma exposure (data from GSK^238^ and our group^244^), probably owing to its high molecular weight and strong lipophilicity (MW = 667, clog*P* = 8.3). Since 2012, GSK has successively patented a series of novel pyrimidone derivatives as Lp‐PLA2 inhibitors.^245^, ^246^, ^247^, ^248^, ^249^, ^250^, ^251^, ^252^, ^253^ Compared with darapladib, these newly reported compounds exhibit a simplified structure, small molecular weight, and low lipophilicity (Figure 11), likely resulting in improved PK properties. Among them, GSK2647544 was a potent and specific inhibitor of Lp‐PLA2 disclosed in an early patent.^245^ In human subjects, GSK2647544 administration was well tolerated for single oral doses up to 750 mg and displayed no significant adverse events.^254^ Remarkably, oral single doses of 125 mg GSK2647544 resulted in improved PK performance compared with single doses of 160 mg darapladib (area under curve [AUC]_(0‐∞)_: 2506 ng·h/mL of GSK2647544 vs 645 ng·h/mL of darapladib, ng·h/mL; *C*_max_: 457 ng/mL of GSK2647544 vs 18 ng/mL of darapladib, ng/mL). In addition, the inhibition of plasma Lp‐PLA2 activity was dose‐dependent, and 80 mg twice per day (160 mg daily dose) inhibited greater than or equal to 80% of plasma Lp‐PLA2 activity at trough, which is necessary for an enzyme inhibitor to produce a clinical effect.^254^ Furthermore, GSK2647544 could likely penetrate into the brains of preclinical species, which was verified by positron emission tomography biodistribution studies using [^18^F] radiolabeled GSK2647544. This clinical trial also found that a twice‐daily dose of 102 mg generated approximately 80% trough inhibition of brain Lp‐PLA2 activity at steady state according to an exploratory analysis.^255^ Taken together, preclinical and preliminarily clinical studies showed that GSK2647544 acted as a useful tool for determining the effects of brain Lp‐PLA2 inhibition. However, another study reported that GSK2647544 might be a moderate to strong CYP3A4 inhibitor, suggesting potential adverse drug‐drug interaction. This finding led to the early termination of this study.^254^ Compounds **19** to **22** are specialized pyrimidine inhibitors with one or two fused heterocycles.^247^ Notably, **19** was reportedly to alleviate the pathological progression of experimental autoimmune encephalomyelitis (EAE) in a preventive treatment model. In addition, ex vivo analysis of treated EAE rats revealed that administration of **19** decreased the levels of proinflammatory M1 markers and increased the markers of anti‐inflammatory M2, indicating the possibility of anti‐inflammation and tissue repair.^246^, ^249^ Recently, GSK disclosed two additional patents based on compounds **21** 251 and **22** 253 together with their own enantiomers. The patent for **21** and its enantiomers emphasized their use in the treatment of neurodegenerative diseases, but no detailed data were offered. Compound **22** was a prodrug containing a monoester of the fumaric acid moiety and maintained the potency of its parent compound **24** against Lp‐PLA2 in vitro (rhLp‐PLA2 IC_50_ = 0.79 nM). Furthermore, **22** significantly improved the solubility of **23** in fasted state simulated intestinal fluid pH 6.5 (FaSSIF) media at room temperature over 24 hours, (857 μg/mL compared with 2.1 μg/mL of parent compound) while displaying favorable stability in three different simulated physiological fluids. Further bioavailability studies in Han Wistar rats demonstrated that **22** could efficiently be converted to compound **23** and that oral dosing at 3 mg/kg **22** resulted in a higher *C*_max_, AUC_(0‐∞)_, and bioavailability of the parent compound compared with oral administration of 3 mg/kg of the parent compound.^253^ Collectively, both the early and later pyrimidone derivatives mentioned above possessed a more than 100‐fold selectivity for Lp‐PLA2 over PLA2‐VIIB. Additionally, the later pyrimidone derivatives released by GSK focused on the identification of novel Lp‐PLA2 inhibitors harboring rational physicochemical properties and desirable PK performance.

**Figure 12:**
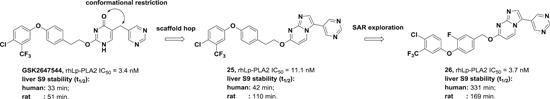
The discovery of a series of imidazo[1,2‐a]pyrimidine compounds as Lp‐PLA2 inhibitors. The IC_50_ values were determined by measuring the rate of turnover of the substrate 2‐thio‐PAF. Assays were performed in 96‐well microtitre plates using recombinant human Lp‐PLA2. Lp‐PLA2, lipoprotein‐associated phospholipase A2; PAF, platelet‐activating factor; SAR, structure‐activity relationship

In the past few years, our group reported two series of novel Lp‐PLA2 inhibitors based on the pyrimidone scaffold, with a favorable selective inhibition toward Lp‐PLA2. Previous studies revealed that GSK2647544 had an improved PK profile compared with darapladib in human subjects.^254^ Indeed, we detected greater plasma exposure of GSK2647544 than that of darapladib when dosed orally to SD rats (GSK2647544: *C*_max_, 0.78 μg/mL; AUC _(0‐∞)_, 3.38 μg·h/mL at 25 mg/kg and darapladib: *C*_max_, 0.15 μg/mL; AUC_(0‐∞)_, 2.11 μg·h/mL at 50 mg/kg). However, GSK2647544 showed a limited elimination half‐life in vivo (*t*_1/2_ = 2.08 hours) and poor metabolic stability (CL_int_ = 74.4 ml/min/kg) in further evaluation with rat liver S9 fractions. Therefore, we utilized a structural restriction strategy that might contribute to greater metabolic stability to identify a new imidazo[1,2‐a]pyrimidine scaffold as an Lp‐PLA2 inhibitor (Figure 12). Subsequently, systematic structure‐activity relationship (SAR) explorations generated a number of Lp‐PLA2 inhibitors with high activity. Compound **26** was selected for further studies of the PK profile and inhibitory activity in vivo. As a result, a longer half‐life (*t*_1/2_ = 13.34 hours) was revealed, which was consistent with good metabolic stability in rat liver S9 fractions and a higher *C*_max_ (2.6 μg/mL) and AUC_(0‐∞)_ (78.1 μg·h/mL). In addition, at a single oral dose of 25 mg/kg, compound **26** could generate over 50% inhibition even 24 hours after administration. The overall inhibition was also comparable to 50 mg/kg darapladib and higher than that for 25 mg/kg GSK2647544. Taken together, these results suggest that the imidazo[1,2‐a]pyrimidine derivatives harbor reasonable physicochemical properties, and are potent, orally bioavailable Lp‐PLA2 inhibitors.^244^

To obtain novel Lp‐PLA2 inhibitors with simplified structures, we also made changes to the pyrimidone scaffold, focusing on the right‐hand side of the structure. Unlike the compounds reported by GSK, we unfolded the fused heterocycle to determine the resulting effects on PK and PD properties. Through three rounds of intensive SAR studies, we obtained numerous inhibitors with high potency against rhLp‐PLA2. Gratifyingly, compound **24** showed decent PK profiles and demonstrated robust inhibitory potency against plasma Lp‐PLA2 in male SD rats after oral administration. Even a relatively low dose of 3 mg/kg led to a greater degree of Lp‐PLA2 inhibition than that of a single oral dose of 25 mg/kg darapladib (Figure 11). Furthermore, **24** significantly inhibited retinal thickening in STZ‐induced diabetic SD rats, a model of DR, after oral dosing for 4 weeks, while darapladib showed similar efficacy at the same oral dose of 25 mg/kg/day. These results further confirm that Lp‐PLA2 is an efficacious therapeutic target for DR.^194^

### 5.3 Biaryl inhibitors

**Figure 13:**
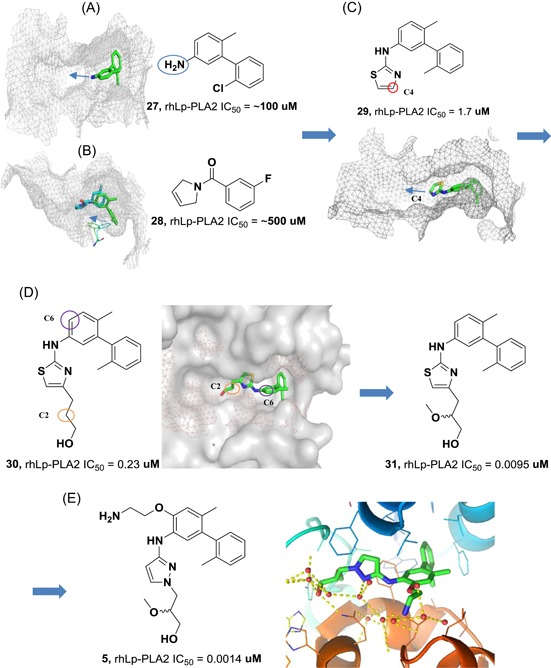
The application of FBLD in the identification of biaryl inhibitors. The binding sites of compounds **27** (PDB: 5JAO) (A), **29** (PDB: 5JAS) (C), **30** (PDB: 5JAT) (D), and **5** (PDB: 5JAU) (E). B, The superimposed binding pockets of compounds **27** (green) and **28** (cyan) (PDB: 5JAL). The binding pockets of compounds **27**, **28**, and **29** are shown in gray meshes. The arrows in each structure indicate that the suitable growth vector of **27** (blue circle), the rotated location of F357 compared with that in the structure of darapladib, and the superior growth vector from C‐4 of thiazole group (red circle). The pocket of compound **30** is shown with gray surface to clarify that C6 of biaryl moiety (purple circle) is facing to the solvent and C2 of *n*‐propyl group (yellow circle) contains an unoccupied pocket. The crystal structure of compound **5** with Lp‐PLA2 is shown in cartoon model. Interacting residues of compound **5** are shown in lines, and inhibitors are rendered in green sticks. The hydrogen bonds are labeled with yellow dotted lines. The figure was prepared using the program PyMOL. The IC_50_ value of compound **28** was determined by measuring the rate of turnover of the substrate PED6, while other compounds were 2‐thio‐PAF. Assays were performed in 96‐well microtitre plates using recombinant human Lp‐PLA2. FBLD, fragment‐based lead discovery; Lp‐PLA2, lipoprotein‐associated phospholipase A2; PAF, platelet‐activating factor; PED6, *N*‐((6‐(2,4‐dinitrophenyl)amino)hexanoyl)‐2‐(4,4‐difluoro‐5,7‐dimethyl‐4‐bora‐3a,4a‐diaza‐*s*‐indacene‐3‐pentanoyl)‐1‐hexadecanoyl‐*sn*‐glycero‐3‐phosphoethanolamine, triethylammonium salt [Color figure can be viewed at wileyonlinelibrary.com]

In primary in vitro assays (fluorescence intensity assays), darapladib showed high activity against rhLp‐PLA2 with an IC_50_ = 0.049 nM. However, the IC_50_ value dramatically declined to 35 nM when assessing the inhibitory potency of darapladib in whole human plasma. To minimize the divergence from the Lp‐PLA2 biochemical and plasma assays, Astex Pharmaceuticals in cooperation with GSK used the FBLD strategy to obtain a potent inhibitor with improved physicochemical properties and speculated that the drop off between the biochemical and plasma assays might result from the high lipophilicity of darapladib. Initially, screening a core fragment set by an Astex Pyramid fragment screen generated 16 X‐ray validated hits. A subset of fragment hits, such as bis‐aryl **27**, were shown to bind to a novel pocket generated by a rotation of the Phe357 side chain, illustrated by an overlay of the binding modes of fragment hits **27** and **28** (Figure 13B). A novel inhibitor that did not interact with the oxyanion hole generated improved physicochemical properties. Together with the suitable growth vector of **27** to an area of the protein where polar interactions were observed in other fragment hits (Figure 13A), the researchers selected **27** as a starting hit. To occupy the binding site of the amino group in amide **28**, compound **29**, with an aminothiazole moiety, was selected for further assessment, primarily due to the superior growth vector from C‐4 and the easier replication of the conformation with nonsulfur‐containing heterocycles (Figure 13C). Under the guidance of the crystal structures of inhibitors bound to Lp‐PLA2, they designed a hydroxypropyl group to occupy a narrow channel formed by Leu153 and Gln352 (compound **30**, Figure 13D), a methoxy group from C‐2 of the *n*‐propyl to fill the small pocket formed by the side chain of Ala355 (compound **31**), and an aminoethoxy moiety from position 6 of the central aryl ring that points toward the solvent to modulate the physicochemical properties and reduce the lipophilicity of the template (compound **5**, Figure 13E). Compound **5** was able to maintain potency and selectivity for Lp‐PLA2, simultaneously with desirable physicochemical properties (MW: 411, Chrom‐Log*D*_pH 7.4_: 3.4, and solubility: 302 μM). In addition, **5** exhibited a lower drop off between the Lp‐PLA2 biochemical (IC_50_ = 0.0014 μM) and plasma assays (IC_50_ = 0.014 μM) relative to darapladib. Notably, these compounds were the first potent Lp‐PLA2 inhibitors that did not directly interact with catalytic residues. However, the work stalled, as neither **31** nor **5** showed satisfactory PK performance consistent with once‐daily dosing in humans. Nonetheless, the identification of novel Lp‐PLA2 inhibitors with improved physicochemical properties and new binding pockets highlighted the success of FBLD in lead identification.^35^

### 5.4 Lactam inhibitors

**Figure 14:**
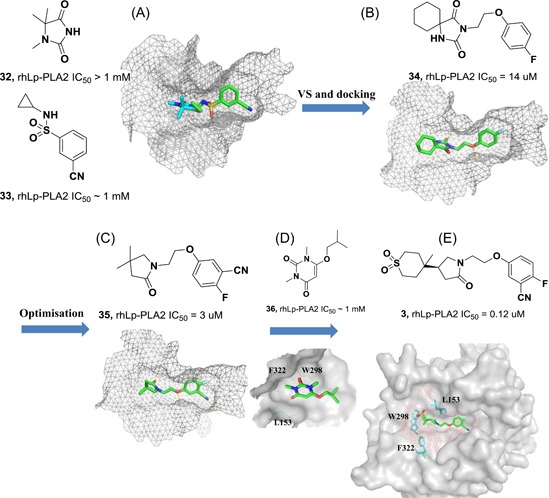
The application of FBLD in the identification of lactam inhibitors. A, The superimposed binding pockets of compounds **32** (cyan) (PDB: 5JAH) and **33** (green) (PDB: 5LZ2). The binding sites of compounds **34** (PDB: 5LYY) (B), **35** (PDB: 5LZ4) (C), **36** (PDB: 5JAN) (D), and **3** (PDB: 5JAS) (E). The superimposed binding pockets of compounds **32** and **33**, and the binding pockets of compounds **34** and **35** are shown in gray meshes. The structures of compounds **36** and **3** are shown in gray surfaces, and the referential interaction from compound **36** to **3** is shown by labeling interacting residues. The hydrogen bonds are labeled with yellow dotted lines. The figure was prepared using the program PyMOL. The IC_50_ value of compound **3** was determined by measuring the rate of turnover of the substrate 2‐thio‐PAF, while other compounds were PED6. Assays were performed in 96‐well microtitre plates using recombinant human Lp‐PLA2. FBLD, fragment‐based lead discovery; Lp‐PLA2, lipoprotein‐associated phospholipase A2; PAF, platelet‐activating factor; PED6, *N*‐((6‐(2,4‐dinitrophenyl)amino)hexanoyl)‐2‐(4,4‐difluoro‐5,7‐dimethyl‐4‐bora‐3a,4a‐diaza‐*s*‐indacene‐3‐pentanoyl)‐1‐hexadecanoyl‐*sn*‐glycero‐3‐phosphoethanolamine, triethylammonium salt; VS, virtual screening [Color figure can be viewed at wileyonlinelibrary.com]

The discovery of biaryl inhibitors suggests that fragment‐based technology could accomplish tighter control of the physicochemical properties of lead compounds that are supportive of further advancements. To obtain novel Lp‐PLA2 inhibitors with improved in vivo parameters during the process of optimization against plasma potency, Astex Pharmaceuticals and GSK conducted a new round of FBLD. Initially, fragment screening gave rise to three fragment hits occupying different regions of the binding site and recapitulated many of the key binding interactions of darapladib (compounds **32**, **33**, and **36**). Although fragment **32** lacked nearly any activity in the biochemical assays (IC_50_ > 1 mM), it was considered an attractive starting point due to the occupancy of the oxyanion hole of Lp‐PLA2 and the excellent vectors for exploring the binding pocket. Another fragment, **33**, was confirmed to bind near the binding pocket of **32**, and merging these motifs with a suitable linker might increase potency according to the result of overlaying their crystal structures (Figure 14A). They then used virtual screening, docking, subsequent X‐ray crystallography, and biochemical assays to screen the in‐house and commercially available compounds containing the hydantoin structure, resulting in the identification of compound **34** as a suitable ligand for further exploration. Subsequent optimization of **34** based on its crystal structure complexed with Lp‐PLA2, led to the development of compound **35** with an improved inhibitory potency (IC_50_ = 3 μM) and good selectivity over PLA2‐VIIB. The potency was also reflected in the crystal structure of **35** in complex with Lp‐PLA2, as the gem‐dimethyl motif tightly filled the groove between the side chains of Leu153, Trp298, and Phe322. The ether oxygen of the linker oriented the phenyl ring into the pocket lined with Phe110, Ala355, and Phe357 and formed close contact with the side chain of Gln352. The cyano completely occupied the small subpocket while forming a long H‐bond with the backbone NH of Phe357. Following the establishment of lactam **35** as the new lead template, growth of compound **35** was then performed by using information from the binding mode of fragment **36** with Lp‐PLA2. Utilizing a suitable substituent that was located in the groove above Trp298 facing the solvent, they identified an alternative low molecular weight, potent, selective inhibitor of Lp‐PLA2 termed compound **3**. The compound showed comparable inhibitory potency to darapladib in whole human plasma assays (**3**, IC_50_ = 32 nM and darapladib, IC_50_ = 35 nM) and the drop off between the Lp‐PLA2 assay and plasma assay was small. Furthermore, PK and safety evaluations demonstrated that compound 3 provided an attractive PK profile and excellent physicochemical properties, suggesting a promising starting point for further lead optimization.^34^

### 5.5 Sulfonamide inhibitors

**Figure 15:**
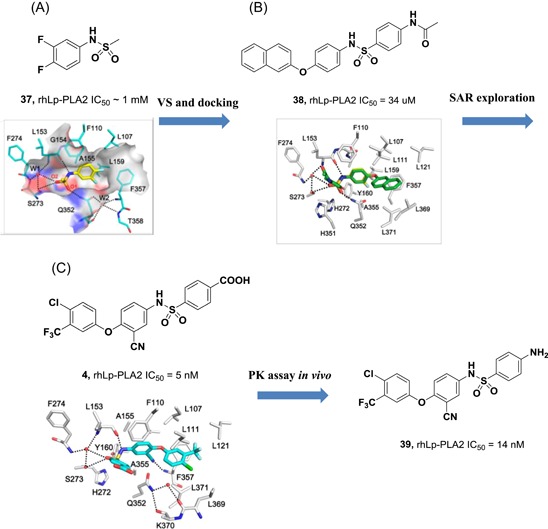
The application of FBLD in the identification of sulfonamide inhibitors. The binding sites of compounds **37** (PDB: 5YE8) (A) and **4** (PDB: 5YEA) (C). B, The superimposed binding pockets of compounds **37** (yellow) and **38** (green) (PDB: 5YE7). The view of fragment **37** within the binding pocket is presented by molecular surface. Black dashed lines represent H‐bonds. **37** (yellow) and the surrounding residues (cyan) are shown as sticks. Two water molecules (W1 and W2) are shown as red spheres. The superimposed binding pockets of compounds **37** (yellow) and **38** (green), together with the binding pocket of compound **4** (cyan), are shown in sticks. Residues interacting with compounds are shown as gray sticks, and black dashed lines represent H‐bonds. The figure was prepared using the program PyMOL. The IC_50_ values were determined by measuring the rate of turnover of the substrate 2‐thio‐PAF. Assays were performed in 96‐well microtitre plates using recombinant human Lp‐PLA2. FBLD, fragment‐based lead discovery; Lp‐PLA2, lipoprotein‐associated phospholipase A2; PAF, platelet‐activating factor; PED6, *N*‐((6‐(2,4‐dinitrophenyl)amino)hexanoyl)‐2‐(4,4‐difluoro‐5,7‐dimethyl‐4‐bora‐3a,4a‐diaza‐*s*‐indacene‐3‐pentanoyl)‐1‐hexadecanoyl‐*sn*‐glycero‐3‐phosphoethanolamine, triethylammonium salt; VS, virtual screening [Color figure can be viewed at wileyonlinelibrary.com]

As previously mentioned, our group disclosed the crystal structures of Lp‐PLA2 in complex with two reversible inhibitors, darapladib and compound **1**, and shed light on the binding mechanisms of different inhibitors with Lp‐PLA2, providing an important prerequisite for the accomplishment of a successful FBLD project.^32^ To enrich the structural types of Lp‐PLA2 inhibitors, we recently reported the discovery of novel sulfonamide inhibitors from a weak binder to a series of nanomolar inhibitors. First, we screened our in‐house 500‐fragment library in PAF enzymatic assays and selected sulfonamide fragment **37** as a promising starting point, which efficiently bound to the oxyanion hole of Lp‐PLA2 (Figure 15A). Second, similarity searching on this fragment followed by molecular docking led to the discovery of a micromolar inhibitor **38** with a 300‐fold potency improvement (**37**, IC_50_ = ~1 mM and **38**, IC_50_ = 34 μM). Superimposing the X‐ray crystal structures of **37** and **38** bound with Lp‐PLA2 showed that compound **38** conserved almost all the aforementioned protein‐ligand interactions of **37**, suggesting a promising hit (Figure 15B). Third, we conducted three rounds of SAR exploration on hit **38**, including filling a subpocket with a cyano group, replacing the naphthyl group that weakly bound to the enzyme with a phenyl group containing both chlorine and trifluoromethyl substitutes, and exploring the hydrophilic substitutions on the solvent‐exposed benzene ring. Ultimately, we obtained compound **4** with an IC_50_ = 5 nM, indicating approximately 200 000‐fold improved affinity from the initial sulfonamide fragment. Subsequently, through preliminary PK assessment in vitro, we identified another sulfonamide inhibitor, **39**, with high inhibitory activity (IC_50_ = 14 nM), decent stability and good permeability. Furthermore, **39** had favorable oral bioavailability in male SD rats (F = 35%) and maintained inhibitory activity for 24 hours post oral administration, which was superior to that of darapladib (F = 11%).^33^

Taken together, using the FBLD strategy, three brand‐new and promising Lp‐PLA2 inhibitors were obtained. These compounds provide a clear example that supports the effectiveness of FBLD for the identification of a good quality lead series. Additionally, learning from these examples may be helpful in the development of fragment‐derived chemotypes with attractive physicochemical properties and PK profiles for other therapeutic targets.

### 5.6 Covalent inhibitors

**Figure 16:**
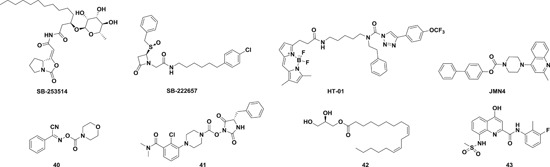
The structures of selected covalent inhibitors and other types of Lp‐PLA2 inhibitors. The IC_50_ values of compounds SB‐253514 and SB‐222657 were determined by measuring the rate of turnover of the artificial substrate DNPG. The IC_50_ values of compounds **40** and **42** were determined by measuring the rate of turnover of the substrate [^3^H] PAF. The IC_50_ values of compounds JMN4 and HT‐01 were determined by using the serine hydrolase‐directed activity‐based probe fluorophosphonate‐rhodamine (FP‐Rh). The IC_50_ value of compound **41** was determined by using HT‐01 as probe. The IC_50_ value of compound **43** was determined by a fluorescent, active‐site‐directed probe AX4870.^267^ DNPG, 1‐decanoyl‐2‐(4‐nitrophenylglutaryl)phosphatidylcholine; Lp‐PLA2, lipoprotein‐associated phospholipase A2; PAF, platelet‐activating factor

Previously, several OP compounds were confirmed by X‐ray crystallography and were able to inhibit Lp‐PLA2 in a covalent manner, including paraoxon, sarin, soman, and tabun, which may be helpful in developing catalytic bioscavengers for OP nerve agents.^64^, ^65^ In addition, other chemicals may covalently bind to Lp‐PLA2 to inhibit its physiological functions (Figure 16). SB‐253514 is a natural product isolated from the culture broths of *Pseudomonas fluorescens* strain DSM11579 and a potent inhibitor of Lp‐PLA2. SB‐253514 could form a 1:1 covalent complex with Lp‐PLA2 confirmed by liquid chromatography‐MS experiments, and further kinetic analysis indicated very slow reactivation (*t*_1/2_ > 24 hours). In addition, SB‐253514 showed good potency against Lp‐PLA2 in isolated rabbit and human plasma and favorable selectivity over other protease enzymes except for PLA2‐VIIB. Furthermore, SB‐253514 significantly inhibited plasma Lp‐PLA2 in WHHL rabbits when dosed intravenously, but no inhibition related to oral administration was observed.^256^ On the basis of the SB‐253514, several cyclic enol‐carbamates were synthesized as potent Lp‐PLA2 inhibitors, but no further information was disclosed.^257^ Unlike SB‐253514, other linear carbamate compounds were also identified as covalent Lp‐PLA2 inhibitors. To obtain tailored activity‐based probes for the detection of Lp‐PLA2 activity in cell and tissue proteomes, Nagano et al^258^ identified a novel class of structurally distinct carbamate Lp‐PLA2 inhibitors by HTS. Among them, JMN4 exhibited superior potency for inhibiting recombinant mouse (IC_50_ = 90 nM) and human (IC_50_ = 5.9 nM) Lp‐PLA2, and excellent selectivity for Lp‐PLA2 over other serine hydrolases, indicating its potential as a qualified probe for studying Lp‐PLA2 function in biological systems.^258^ According to a model of inhibition of Lp‐PLA2 by JMN21 (structure not shown) optimized from JMN4, this series of Lp‐PLA2 inhibitors may form a covalent adduct with the active‐site Ser273, similar to that of the OP complexes.^259^ Monocyclic‐β‐lactams, such as SB‐222657, were reported to generate a covalent modification of the Lp‐PLA2 active site. The calculated value of *K*_i_ for SB‐222657 was 40 nM, while it was only weakly active on lecithin:cholesterol acyltransferase and completely inactive against paraoxonase. In addition, SB‐222657 was used as a tool to determine the role of Lp‐PLA2 in the dysregulated phospholipid metabolism that occurs during oxidative modiﬁcation of lipoproteins, suggesting promising value.^260^, ^261^

Three additional inhibitors likely induced a covalent modification in Lp‐PLA2 though this requires confirmation. After screening 4480 compounds and performing SAR studies, Jeong et al improved a weakly active hit (IC_50_ = 3.8 μM) containing an oxime moiety to a highly potent compound **40** (IC_50_ = 50 nM), based on the whole human plasma assay under identical conditions.^262^, ^263^ Compound HT‐01 was likely to bind to Lp‐PLA2 in a covalent manner and was first identified as an activity‐based probe for another serine hydrolase DAGLβ. In addition to the inhibition of mouse Lp‐PLA2 with an IC_50_ = 39 nM, HT‐01 could serve as a tailored activity‐based probe for the detection of this enzyme in native proteomes according to gel‐based activity‐based protein profiling assays.^264^ Recently, Abide Therapeutics disclosed a patent for a series of dual inhibitors of both Lp‐PLA2 and α/β‐hydrolase domain containing 6 (ABHD6). As an example, compound **41** possessed a carbamate moiety and could efficiently inhibit 50% of Lp‐PLA2 and ABHD6 at concentrations lower than 100 nM.^265^ However, although these noncanonical carbamates may provide a covalent resource to Lp‐PLA2, their actual binding modes require verification in future studies.

### 5.7 Other inhibitors

Compound **42** is natural fatty acid glycerol isolated from *Saururus chinensis* roots that showed narrow inhibitory activity against Lp‐PLA2 with an IC_50_ value of 45 μM (Figure 16). The amides of xanthurenic acid were other specific scaffolds of Lp‐PLA2 inhibitors.^266^ Initially, Lin et al^267^ identified novel inhibitors of Lp‐PLA2 with amides of the xanthurenic acid moiety, and AX10185 had an IC_50_ value of 27 nM in human plasma assays. Subsequent SAR optimization led to the identification of compound **43** with rhLp‐PLA2 and plasma Lp‐PLA2 IC_50_ values of 3 and 9 nM, respectively. Interestingly, these derivatives inhibited Lp‐PLA2 in a metal‐dependent manner. With the addition of ZnCl_2_, the IC_50_ value gradually decreased to a steady number. In addition to being useful for the development of novel metal‐dependent Lp‐PLA2 inhibitors, the work also emphasized the importance of considering the influence of metals on the enzyme/receptor binding of small molecules.^268^ Furthermore, the results also highlighted quinolone‐metal chelators as potential enzyme inhibitors.

To summarize, although a number of Lp‐PLA2 inhibitors can be used at present, the aforementioned compounds have been used sparingly with the exception of pyrimidinone derivatives. Seemingly, the major factor that limits further exploration is the difficulty in balancing the inhibitory potency and physicochemical properties. As mentioned above, Lp‐PLA2 is a hydrophobic protein, and its hydrophobic interaction mainly contributes to the affinity between the ligand and protein. However, high lipophilicity is not preferable for an eligible drug candidate, as lower lipophilicity inhibitors (clog*P* < 5) are associated with decreased attrition in clinical trials. In this regard, FBLD appears to be a favorable choice, because it can sensitively detect promising fragments from small libraries (approximately 1000 compounds) consisting of low molecular weight and relatively polar fragments (for example, heavy atom count ≤16 and a mean clog*P* of 1.4), and efficiently develop them into desirable lead molecules with the aid of various technical means. Indeed, this favors the discovery of novel Lp‐PLA2 inhibitors with favorable physicochemical properties. Inspired by the successful application of FBLD, we can facilitate the development of Lp‐PLA2 inhibitors by introducing other emerging technologies.^34^, ^69^

## 6 DISCUSSION AND PROSPECTS

Approximately 40 years ago, Farr et al reported the presence of an acid‐labile factor that rapidly inactivated PAF in human serum, later referred to as pPAF‐AH.^269^, ^270^ They further determined various aspects of its nature, including selectivity for PAF, lack of calcium dependence, presence in LDL, and inhibition by defluorinated phosphate (DFP).^271^ Due to its ability to exhibit phospholipase A2 characteristics and circulate in the blood via binding to LDL and HDL, pPAF‐AH is also called Lp‐PLA2. Subsequently, Stafforini et al made great efforts to determine the biological properties and functions of Lp‐PLA2. Since those studies, the effects of Lp‐PLA2 on inflammatory processes have been widely studied, mainly due to the ability of inflammatory cells, especially macrophages, to secrete the majority of circulating Lp‐PLA2, particularly, during the differentiation of monocytes to macrophages, indicating the occurrence of inflammation.^24^, ^25^, ^60^ Coupled with a high distribution of Lp‐PLA2 in the blood, the association between Lp‐PLA2 and vascular inflammatory‐related diseases, such as atherosclerosis, DR, AD, and certain cancers,^19^, ^20^, ^21^, ^22^, ^195^ has been evaluated. In this context, several compounds with diverse scaffolds have been identified as potent Lp‐PLA2 inhibitors, providing several excellent probe molecules. In addition, benefiting from the FBLD strategy, recently reported Lp‐PLA2 inhibitors exhibit favorable physicochemical properties and PK profiles.^34^, ^35^ In terms of their clinical applications, although Lp‐PLA2 inhibitors failed to reduce the incidence of CVD events, they indeed showed significant therapeutic effects on microvascular diseases such as DR and AD. Nonetheless, we could not ignore the neutral and negative reports regarding Lp‐PLA2 inhibition, which may be attributed to its anti‐inflammatory roles in some conditions. To date, the effects of Lp‐PLA2 on various diseases remain inconsistent. Therefore, to motivate further research within the Lp‐PLA2 field, we would like to offer several personal insights into the achievements from the past few decades in the following text.

### 6.1 Genetic studies

GSK has heavily invested in the development of Lp‐PLA2 inhibitors, particularly, into two unsuccessful phase III trials. Several hundreds of million dollars were spent and over 30 000 patients were recruited and followed up for a number of years. These failures not only dimmed the future of the anti‐inflammatory strategy in atherosclerosis drug research, but also led to significant financial losses to the company. Genetic studies, particularly, those of rare loss‐of‐function variants, have been considered a promising approach to promote the development of targeted drugs by validating or invalidating novel therapeutic targets. Recently, two large‐scale genetic studies established that *PLA2G7* variants that reduced Lp‐PLA2 activity to levels comparable to darapladib had no effect on the risk of CHD and outcomes. These results deny, to a certain extent, a causal role of Lp‐PLA2 during atherosclerotic progression and are consistent with an early genetic study on *PLA2G7* variants that resulted in a modest reduction in Lp‐PLA2 activity.^77^, ^89^, ^114^ Therefore, we speculate that a comprehensive genetic study before the clinical trial may be conducive to the identification of the fundamental disease pathways. In addition, genetic studies contribute to differentiating between responding and nonresponding patients. Very recently, a pharmacogenetic meta‐analysis identified a potentially efficacious subgroup within the large global trials of darapladib. Among them, one common variant (rs181937009) conferred risk in the placebo arm but reduced the risk in the darapladib arm, suggesting of an improved outcome with the minor allele following treatment with darapladib compared with placebo.^38^ Similarly, another SNP of *PLA2G7* (rs1805018) was reported to affect plasma TG levels in response to supplementation with fish oil‐derived long‐chain n‐3 PUFAs, signifying the interindividual variability in plasma TG levels following n‐3 PUFA supplementation.^122^ Furthermore, ischemic stroke patients carrying the rs1051931 and rs7756935 variants (AA‐CC haplotype) were found to have a considerably higher risk of aspirin resistance than noncarriers (OR = 8.23, 95% CI: 1.59‐42.63).^121^ Accordingly, through the determination of the consequences of mutations, particularly, loss‐of‐function variants, within potential therapeutic target genes by systematic and large‐scale human genetic studies, predicting the potential efficacy and safety of the compounds aimed at these targets is possible. Since impressive advances in DNA analysis technology aid the implementation of systematic and large‐scale genetic studies, we need to take advantage of the data mining opportunities generated from the clinical trials to make full sense of the consequences of Lp‐PLA2 inhibition.

### 6.2 Nominal “safety window”

Divergence seems to be ubiquitous among Lp‐PLA2 studies, including the results of epidemiologic studies, the impacts of genetic mutations, and the role of Lp‐PLA2 in inflammation and disease progression. The causes of divergence are manifold, including complex experimental systems, different objects under study, and inconsistent methods of measurement and analysis. Nevertheless, we speculate that the contradiction of Lp‐PLA2 in modulating inflammation might be a key causative factor for these divergences. Both the substrates (PAF and oxLDL) and products (lysoPC) of Lp‐PLA2 hydrolytic action were demonstrated to be proinflammatory mediators.^13^, ^148^ Consequently, the accumulation of any of these mediators resulting from a fluctuation in Lp‐PLA2 activity could theoretically promote the inflammation process. Apparently, Lp‐PLA2 expression should lie within a rational “window” to maintain body homeostasis. Indeed, Stafforini^60^ proposed an “expression window” of Lp‐PLA2 that is a key determining factor in response to endotoxemia, based on the dynamics of Lp‐PLA2 in patients most with severe sepsis and other critical illnesses. In this case, the survivors could maintain Lp‐PLA2 activity within a range comparable to that of healthy controls, while the nonsurvivors displayed significant deviation from this “expression window” in either direction.^272^ In fact, we agree with this concept to a degree, as we believe in the existence of the top edge of such a window but not the bottom edge. We hypothesize that extended exposure to a high level of Lp‐PLA2 activity may result in the development of various diseases, while inadequate or even absent Lp‐PLA2 activity may not be detrimental. The primary basis for our hypothesis might be that Lp‐PLA2 inhibition showed limited impacts on the accumulation of PAF and oxLDL but significantly decreased the lysoPC level, suggesting a dominant proinflammatory effect of Lp‐PLA2.^50^, ^51^, ^273^ Furthermore, the loss‐of‐function variants of Lp‐PLA2 were confirmed not to be associated with certain diseases in large‐scale studies.^76^, ^77^ Moreover, in the clinical trials, serious adverse effects associated with intensive Lp‐PLA2 inhibition by darapladib or rilapladib were similar to those in the placebo group, indicating the absence of target‐specific toxicity.^19^, ^20^, ^21^, ^22^ Altogether, we attribute the detrimental consequences to an elevated Lp‐PLA2 level. Since the direct receptor‐mediated signaling events of Lp‐PLA2 were not demonstrated, its biological effects are tightly associated with the upstream and downstream molecules. In this regard, more attention should be paid to Lp‐PLA2‐derived lysoPC, as it accumulates with an increase in Lp‐PLA2. Furthermore, lysoPC, particularly the 16:0 lysoPC, a main product of Lp‐PLA2, was associated with several diseases, including NASH,^228^ DR,^37^ CAVD,^274^ and CNS diseases.^14^ Therefore, we assume that alterations in the lysoPC level, the ratio between Lp‐PLA2 and lysoPC, or even the proportion among Lp‐PLA2, lysoPC, and oxLDL may be an alternative monitoring metric for determining the role of Lp‐PLA2 in various diseases. In addition, we anticipate that the multifactor evaluation can diminish the occurrence of inconsistent results among studies.

### 6.3 Biological functions

Although certain progress has been achieved, the biological functions of Lp‐PLA2 require further investigation due to conflicting results being reported. Moreover, the scope of Lp‐PLA2 studies should be expanded from the blood to other potential secretory tissues. Recently, Jackisch et al^41^ revealed that human adipose tissue appeared to be an active source of Lp‐PLA2 and was differentially regulated by fat depot and metabolic status. For example, the abdominal subcutaneous but not the omental, adipose tissue displayed enhanced Lp‐PLA2 expression, and type 2 diabetes status contributed to the upregulation of Lp‐PLA2 in adipose tissue. Owing to its association with inflammation and atherosclerotic risk, Lp‐PLA2 action within adipocytes may represent a potential therapeutic target to prevent the development of cardiometabolic complications in type 2 diabetes.^41^ On the basis of this study, the perivascular adipose tissue (PVAT), which surrounds most large blood vessels, is probably a potential source of Lp‐PLA2 as well. Moreover, the role of PVAT in aortic aneurysms and vasculitic syndromes was demonstrated. Therefore, determining the biological functions of adipose‐derived Lp‐PLA2 is essential.^275^, ^276^ In addition, the level of Lp‐PLA2 derived from the brain tissue remains unclear. Earlier, Cao et al^123^ determined that Lp‐PLA2 mRNA was expressed in all regions of the brain,^123^ while a recent study failed to detect Lp‐PLA2 expression within autopsy brain tissue through histopathologic analyses.^184^ Moreover, rilapladib is not considered to be a brain penetrant based on the preclinical data of GSK and that of our group but shows therapeutic effects on AD, suggesting a mediating mechanism by peripheral tissue.^21^ Therefore, whether brain tissue can secrete Lp‐PLA2 and what the biological functions of brain‐derived Lp‐PLA2 are remain to be established. In addition to the source, the pathophysiological role and clinical significance of Lp‐PLA2 bound to LDL and HDL should be determined. HDL‐associated Lp‐PLA2 is highly acknowledged to be anti‐inflammatory, while LDL‐associated Lp‐PLA2 is proinflammatory; however, the underlying mechanisms have yet to be elucidated. Alterations in the distribution of Lp‐PLA2 between LDL and HDL were detected in patients with various diseases, including atherosclerosis, dyslipidemias,^12^ DM,^277^ and PCOS.^278^ However, the clinical consequences of these alterations were inadequately determined.^12^ Furthermore, no data are available regarding the effects of selectively inhibiting LDL‐ or HDL‐associated Lp‐PLA2, as almost all published Lp‐PLA2 inhibitors focus on inhibition of total plasma Lp‐PLA2 activity, which primarily concerns LDL‐associated Lp‐PLA2. Furthermore, the pathological role of Lp(a) has gradually attracted more attention, especially in CVD. Since Lp‐PLA2 was identified as an important determinant of Lp(a) functionality and pathophysiology, the potential of Lp(a)‐associated Lp‐PLA2 as a therapeutic target needs to be elucidated.^12^, ^221^, ^222^

### 6.4 Inhibitor discovery

The discovery of inhibitors of a hydrophobic protein is challenging, as it is difficult to balance the potency and physicochemical properties. The FBLD strategy assists us in addressing this issue.^69^ The successful application of FBLD to the identification of novel Lp‐PLA2 inhibitors provides a practical example for the discovery of drugs for other targets.^33^, ^34^, ^35^ Furthermore, the possibility of developing LDL‐Lp‐PLA2‐ or HDL‐Lp‐PLA2‐specific inhibitors using the FBLD strategy or other emerging technologies are worthy of investigation. Additionally, to determine the efficacy accurately, various diseases may require the tested compound to have specific properties; thus extending the structural types of Lp‐PLA2 inhibitors is necessary.

In summary, Lp‐PLA2‐targeted drug development has always been full of controversy from the beginning, due to the rather complicated functions of the enzyme and its considerable involvement in a wide variety of diseases. The “door” to atherosclerosis was almost shut after the failure of darapladib in phase III clinical trials, while a “window” seemed to be opened for microvascular diseases. Clinical and preclinical studies not only provided basic evidence for the efficacy of Lp‐PLA2 inhibitors in the treatment of AD and DME but also elucidated specific mechanisms of action that were independent of the most studied risk factors, such as Aβ and VEGF. In view of the high attrition rate of Aβ‐based anti‐AD drugs and the poor compliance of DME patients for anti‐VEGF biologics, Lp‐PLA2 inhibitors can yet be regarded as a potential good choice. With a series of eligible compounds available at present, scientists would be admired if they exerted more effort in exploring the role of Lp‐PLA2 in diseases other than atherosclerosis, particularly, in microvascular diseases that may be more sensitive to Lp‐PLA2 inhibition. In addition, the large pool of data generated from clinical trials provides numerous opportunities for comprehensive data mining, which may be beneficial to understanding the biological consequences of Lp‐PLA2 inhibition and help us to learn a profound and valuable lesson from the failure of darapladib.^279^, ^280^, ^281^, ^282^, ^283^, ^284^, ^285^, ^286^, ^287^, ^288^ To conclude, we anticipate that the story of Lp‐PLA2 will continue.
